# Defense responses of lentil (*Lens culinaris*) genotypes carrying non-allelic ascochyta blight resistance genes to *Ascochyta lentis* infection

**DOI:** 10.1371/journal.pone.0204124

**Published:** 2018-09-20

**Authors:** Ehsan Sari, Vijai Bhadauria, Larissa Ramsay, M. Hossein Borhan, Judith Lichtenzveig, Kirstin E. Bett, Albert Vandenberg, Sabine Banniza

**Affiliations:** 1 Department of Plant Sciences/Crop Development Centre, University of Saskatchewan, Saskatoon, Saskatchewan, Canada; 2 Agriculture and Agri-Food Canada, Saskatoon Research and Development Centre, Saskatoon, Saskatchewan, Canada; 3 School of Agriculture and Environment, University of Western Australia, Perth, Western Australia, Australia; New South Wales Department of Primary Industries, AUSTRALIA

## Abstract

Ascochyta blight of lentil is an important fungal disease in many lentil-producing regions of the world causing major yield and grain quality losses. Quick shifts in aggressiveness of the population of the causal agent *Ascochyta lentis* mandates developing germplasm with novel and durable resistance. In the absence of complete resistance, lentil genotypes CDC Robin and 964a-46 have frequently been used as sources of partial resistance to ascochyta blight and carry non-allelic ascochyta blight resistance genes. RNA-seq analysis was conducted to identify differences in the transcriptome of CDC Robin, 964a-46 and the susceptible check Eston after inoculation with *A*. *lentis*. Candidate defense genes differentially expressed among the genotypes had hypothetical functions in various layers of plant defense, including pathogen recognition, phytohormone signaling pathways and downstream defense responses. CDC Robin and 964a-46 activated cell surface receptors (e.g. receptor like kinases) tentatively associated with pathogen-associated molecular patterns (PAMP) recognition and nucleotide-binding site leucine-rich repeat (NBS-LRR) receptors associated with intracellular effector recognition upon *A*. *lentis* infection, and differed in their activation of salicylic acid, abscisic acid and jasmonic acid / ethylene signal transduction pathways. These differences were reflected in the differential expression of downstream defense responses such as pathogenesis-related proteins, and genes associated with the induction of cell death and cell-wall reinforcement. A significant correlation between expression levels of a selection of genes based on quantitative real-time PCR and their expression levels estimated through RNA-seq demonstrated the technical and analytical accuracy of RNA-seq for identification of genes differentially expressed among genotypes. The presence of different resistance mechanisms in 964a-46 and CDC Robin indicates their value for pyramiding gene leading to more durable resistance to ascochyta blight.

## Introduction

Ascochyta blight caused by the fungal pathogen *Ascochyta lentis* Vassilievsky (teleomorph: *Didymella lentis* W.J. Kaiser, B.C. Wang, and J.D. Rogers) is a major foliar disease of lentil in temperate regions of the world. Symptoms include lesions on aerial parts of the plant including leaf, stem and pod, and yield losses of up to 70% have been reported in Canada [1]. Use of resistant varieties is the most effective control measure; however, crop rotation, use of disease-free seeds and fungicides are also recommended. Lentil varieties with partial resistance to ascochyta blight have been developed [2–4]. When studying the pathogenic variability among 100 isolates of *A*. *lentis* collected from Canada and 13 other countries, Ahmed et al. [5] found that aggressiveness of isolates had increased over time as isolates collected in 1978 and 1985 showed less virulence than those collected in 1992. The Canadian cultivar ‘Laird’ which was moderately resistant at the time of release in the 1980s, has become susceptible as a result of increase in the aggressiveness of isolates [5]. Davidson et al. [6] reported an increase in the aggressiveness of the *A*. *lentis* population in southern Australia when isolates collected from 2005 to 2014 were compared. The rapid shift in the aggressiveness of *A*. *lentis* requires developing durable resistance through pyramiding of multiple resistance genes, preferably those that mediate resistance through different mechanisms.

Plant innate immunity responses could typically be described as a two tiered and interconnected defense response [7]. The first line of plant defense is triggered when pathogen-associated molecular patterns (PAMP) are perceived by the trans-membrane pattern recognition receptors (PRRs), which has been termed PAMP-triggered immunity (PTI). PAMPs are conserved, slowly-evolving molecules and mutations in genes encoding PAMPs are usually detrimental for the pathogens. Examples of well-known PAMPs are flagellin, cold shock proteins, elongation factors in bacteria and chitin in fungi [8]. PRRs are usually receptor-like protein kinases (RLK) with a trans-membrane domain such as the putative chitin receptor LysM/CERK1 [9], peptide receptors [10], the oligogalacturonides receptor wall-associated kinase 1 (WAK1) [11] and brassinosteroid insensitive 1-associated kinase 1 (BAK1) [12]. Resistance induced by PTI is quantitative and effective against all pathogens regardless of their life-style [13]. Plant pathogens overcome PTI by secreting into the host an array of small molecules and proteins known as pathogenic effectors. The second layer of plant defense involves the effector-triggered immunity (ETI) caused by the perception of the effectors by plant disease resistance (R) proteins. ETI is faster and stronger than PTI, and usually leads to a form of plant cell death known as hypersensitive response (HR). Most R proteins are nucleotide-binding site leucine-rich repeat (NBS-LRR) proteins. Based on their N-terminal domain, plant R proteins are classified as toll/interleukin-1 receptor (TIR)-NBS-LRR or coiled-coil (CC)-NBS-LRRs [14].

Three pathogen classes are recognized including biotrophs, necrotrophs and hemibiotrophs and according to their mode of nutrition acquisition, the kinetics, components and outcomes of the PTI and ETI response are broadly determined [13]. Biotrophs are dependent on living cells for acquiring nutrients. A typical resistance mechanism against biotrophs is perception of effectors by host plant R proteins leading to HR and their deprivation of the food sources [15]. Mechanisms of plant defense against hemibiotrophs and necrotrophs are distinct from those against biotrophs and vary with plant species and virulence mechanisms of pathogens [13]. Plant hormones play an important role in modulating the defense response against various classes of pathogens and their contributions vary accordingly. Most defense responses effective against necrotrophs are activated by the phytohormones ethylene (ET) and jasmonic acid (JA), whereas salicylic acid (SA) primarily regulates resistance to biotrophs and some hemibiotrophs [13]. However, a possible role of the salicylic acid (SA) signaling pathway and systemic acquired resistance (SAR) in defense against the model necrotroph *Botrytis cinerea* was reported for *Arabidopsis thaliana* plants impaired in the SA signaling pathway [16]. The role of abscisic acid (ABA) in defense against necrotrophs is controversial and both augmented resistance and susceptibility to pathogens have been reported as evident from the response to diseases in ABA-deficient mutants. The ABA pathway triggers multifaceted defense responses in plants which vary with the type of plant tissues, the infection stage and the strategy of the pathogens [17]. For example, a mutation in the ABA pathway in the tomato mutant *sitiens* showed increased resistance to the necrotroph *B*. *cinerea* through increasing the accumulation of reactive oxygen species (ROS) [18,19]. By contrast, callose deposition mediated by the ABA signaling positively contributed to the resistance of *A*. *thaliana* to the hemibiotroph *Leptosphaeria maculans* [20]. Other phytohormones such as gibberellic acid [21] and auxin [22] are also involved in defense signal transduction against necrotrophs. Accumulation of each or a blend of these phytohormones induces the activation of downstream defense responses involved in cell-wall reinforcement, accumulation of ROS, and the synthesis of pathogenesis-related (PR) proteins and antimicrobial secondary metabolites via the phenylpropanoid pathway [13,23].

The mechanisms of lentil resistance to *A*. *lentis* were previously investigated in resistant genotype ILL 7537 and compared to susceptible genotype ILL 6002 using the ‘Pulsechip’ microarray which was developed mostly using sequences of several relative legume species [24]. Results indicated substantial differences in type, level and activation time of genes differentially expressed upon *A*. *lentis* challenge in the resistant and susceptible genotypes. This partially explained mechanism of ascochyta blight resistance in genotype ILL 7537. Due to the limited number of genes that could be assessed, sequence dissimilarity between lentil and related legume species and the technical drawbacks of microarrays, this approach was not sensitive enough to fully describe the lentil transcriptome expressed in response to *A*. *lentis* infection. Garcia et al. [25] conducted a SuperSAGE transcriptome analysis of responses to *A*. *lentis* in lentil genotype ILL 5588. Genes differentially expressed after *A*. *lentis* infection were annotated as disease resistance genes (31 transcripts), transcription factor (66 transcripts) and kinases (197 transcripts). More recently, transcriptome profiling of ILL7537 and ILL 6002 genotypes during the first 24 h after *A*. *lentis* infection suggested earlier and faster defense responses in ILL 7537 than ILL 6002 genotype [26]. These studies have either capitalized on the sequences of legume relative species [24] or on *de novo* assembly of transcripts [25,26]. The availability of a lentil genome [27] has simplified the transcriptome analysis by reducing the complexity of analysis, decreasing the alignment biases and reducing redundancy (reads of the same gene are not grouped in one cluster) and chimerism errors (reads of the different genes are grouped in one cluster) associated with *de novo* transcriptome analysis [28].

Histological differences in the reaction to *A*. *lentis* infection and induction of the two major defense signaling pathways employing SA and JA were reported for the lentil genotypes CDC Robin and 964a-46, which carry non-allelic resistance genes for partial resistance to ascochyta blight in comparison with the susceptible check Eston [29]. This study indicated that cell death inhibition is possibly a mechanism of resistance in CDC Robin, whereas 964a-46 underwent cell death similar to the susceptible control Eston. The ascochyta blight resistant genotypes CDC Robin and 964a-46 differed in their expression of genes associated with the SA and JA signaling pathways. Infection by *A*. *lentis* led to an intensive induction of the SA-related genes only in 964a-46. The expression of genes associated with the JA pathway was associated with the levels of ascochyta blight resistance in CDC Robin and 964a-46, since CDC Robin, with earlier and higher induction of the JA pathway, showed higher levels of resistance to ascochyta blight than did 964a-46 [29]. Considering these substantial differences, pyramiding resistance genes of CDC Robin and 964a-46 genotypes, in theory, should lead to more durable resistance to ascochyta blight. However, a more detailed study of the defense mechanisms through RNA-seq analysis to gain more detailed knowledge of upstream and downstream gene expression in both accessions was warranted. The present study was conducted to gain detailed knowledge of gene expression profiles and identify potential candidate resistance genes for further investigation.

## Materials and methods

### Plant materials and *Ascochyta lentis* inoculation

CDC Robin [4] and 964a-46 are lentil lines used as sources of ascochyta blight resistance at the Crop Development Centre (CDC), University of Saskatchewan, Canada. The partial resistant to ascochyta blight of CDC Robin was derived from cv. Indianhead [30]. Ascochyta blight resistance in 964a-46 was derived from ILL 5588, which is also the source of resistance for Australian cultivar Northfield [2]. Lentil cv. Eston was used in this study as a susceptible check [30]. *A*. *lentis* isolate AL57 is an aggressive isolate from Landis, Saskatchewan [31]. A conidial suspension prepared from a monoconidial culture of AL57 was stored at -80°C in a cryopreservation solution containing 10% skim milk and 20% glycerol. To prepare fungal inoculum, conidia were revitalized on 50% oatmeal agar plates (30 g oatmeal [Quick Oats, Quaker Oats Co., Chicago, IL, USA], 8.8 g agar [Difco, BD^®^, Sparks Glencoe, MD, USA], 1 L H_2_O) and incubated for 7 d at room temperature. The spore suspension was prepared following the protocol described by Vail and Banniza [32]. The concentration of the suspension was adjusted to 10^6^ conidia mL^-1^ using a hemocytometer.

Four seeds of each genotype were sown in 10 cm square pots containing a soilless mixture of Sunshine Mix No. 4 (Sun Grow Horticulture^®^ Ltd., Vancouver, BC, Canada) and Perlite^™^ (3/1 V/V). Three pots serving as biological replicates were assigned to each of eight sampling time points 0 (mock inoculated and sampled before inoculation), 6, 12, 18, 24, 36, 48, 60 h post inoculation (hpi). Pots were maintained in a greenhouse with average daily temperate of 23.5 °C, relative humidity of 66% and a light regime of 18/6 h day/night supplied from natural light supplemented with an artificial light source in the form of 1000 watts high pressure sodium lights. Seedlings were inoculated with the conidia suspension at a rate of 2 mL per seedling, which was equivalent to run-off, using an airbrush 21 d after sowing, and were incubated in a humidity chamber for 48 h in dark. Plants were then incubated on a misting bench, receiving mist for 30 s every 90 min during the day (approximately 12 hrs) for the remainder of the test. The experiment was conducted as a randomized complete block design.

### Illumina sequencing and RNA-seq analysis

All inoculated leaflets of seedlings assigned to each biological replicate of different sampling time points were collected and flash frozen in liquid nitrogen. Leaflets pooled for each biological replicate were ground in an RNAse free mortar, pre-cooled with liquid nitrogen and samples from one biological replicate was subjected to RNA sequencing. RNA was extracted using a combination of the standard Trizol^®^ reagent (Invitrogen, Carlsbad, USA) protocol and an Ambion^®^ PureLink^™^ RNA mini kit with on-column PureLink^®^ DNAse treatment (Invitrogen, Carlsbad, USA) following the manufacturer’s instructions. The purity of RNA was tested using a NanoDrop ND8000 (Thermo Scientific, Wilmington, USA) and samples with an A260/280 ratio less than 2.0 were discarded. The quantity of RNA was determined using a Qubit^®^ 2.0 Fluorometer (Grand Island, NY, USA) and a Qubit^™^ RNA broad range assay kit (Invitrogen, Carlsbad, USA) following the manufacturer’s protocol. The integrity of RNA was determined using an Agilent 2100 Bioanalyzer (Agilent Technologies Inc., Santa Clara, USA).

Library preparation and Illumina sequencing was performed at the National Research Council (NRC) Nucleic Acid Sequencing Laboratory, Saskatoon, Canada. Total RNA (~1 μg) for each sample was used for library preparation using Illumina TruSeq^®^ RNA sample preparation v. 2 kit (Illumina, San Diego, USA). The samples were then sequenced (2 ×101 cycles, paired-end reads) on the HiSeq 2500 (Illumina, San Diego, CA, USA) using the TruSeq SBS v3-HS 200 cycles Kit (Illumina, San Diego, CA, USA).

Resulting reads were filtered to retain only those with a Phred quality score of greater than 30 and a length of at least 25 nucleotides using Prinseq v 0.20.4 [33]. The remaining paired-reads were mapped to the *A*. *lentis* genome using the spliced read mapper software TopHat 2.0.7 (https://academic.oup.com/bioinformatics/article/25/9/1105/203994) to classify the transcripts as plant- or pathogen-derived. The *A*. *lentis* genome sequence used was of the Australian isolate Al4 [6], assembly version 130419. Processed reads were extracted from TopHat BAM files using Picard v1.95 (https://broadinstitute.github.io/picard/) for mapped reads and BamUtils v 1.0.5 [34] for unmapped reads.

RNA-seq analysis was conducted on the iPlant Collaborative^™^ server (Arizona Genomic Institute, Tucson, USA). The total number of high quality plant-derived paired-end reads generated by Illumina sequencing was 72,528,007 for Eston, 63,031,665 for CDC Robin and 69,060,713 for 964a-46 (S1 Table). The plant derived paired-end reads were deposited in the Sequence Read Archive of the National Center for Biotechnology Information (NCBI; SRA accession no. SRP127015, BioProject no. PRJNA422815). Plant derived paired-end reads were mapped to the lentil draft genome v 0.6 of cultivar CDC Redberry [27] using TopHat v. 2.0.9 (https://academic.oup.com/bioinformatics/article/25/9/1105/203994). The mean percentage of paired-end reads mapped to CDC Redberry sequences was 90.7% for Eston, 91.0% for CDC Robin and 90.5% for 964a-46, and 83.8% of reads were uniquely mapped to the reference genome for Eston, 83.4% for CDC Robin and 82.7% for 964a-46 (S1 Table). The mean percentage of total paired-end reads mapped to the *A*. *lentis* reference genome was 0.3 for Eston, 0.2 for CDC Robin and 0.3 for 964a-46. Transcripts were assembled for Eston, CDC Robin and 964a-46 with the software Cufflinks (http://cole-trapnell-lab.github.io/cufflinks/) using the reference genome sequences and BAM files generated from TopHat. The total number of transcript was 23,663 for Eston, 22,789 for CDC Robin and 24,398 for 964a-46. Pearson’s correlation analysis was conducted for the gene expression values of samples used in the RNA-seq analysis with the hierarchical clustering tool of CLC Genomics Workbench^®^ 7.0.3 (https://www.qiagenbioinformatics.com/products/clc-genomics-workbench/) using the average linkage distance among samples following the statistical procedure suggested by Eisen et al. [35].

Gene expression values normalized using fragments per kb of exon per million mapped reads (FPKM) were used for calculation of fold change in expression levels by dividing the FPKM value of infected samples to that of 0 hpi using Cuffdiff (http://cole-trapnell-lab.github.io/cufflinks/cuffdiff/). The exon lengths used for the FPKM calculation were the lengths of putative transcripts identified by Cufflinks obtained after mapping the paired-end reads to the reference genome for each genotype. A relative expression fold change of two was considered as a threshold for identifying differentially expressed genes.

The expression levels of a few housekeeping genes including *glyceraldehyde 3-phosphate dehydrogenase* (*GAPDH*), α and β-*tubulin*, *DNAj Chaperon* and *transcription elongation factor* (*TEF*) were extracted from the Cuffdiff output (S2 Table). Differences in the expression of the housekeeping genes were subtle across different sampling time points, conforming to the rule that pathogen infection does not modify the expression of housekeeping genes, and is an indicator of lack of technical errors within samples collected over time.

To identify functional descriptions for up-regulated genes, the corresponding genomic sequences of CDC Redberry were extracted and subjected to reciprocal BLASTx analysis using standalone BLASTx v. 2.2.29 (https://blast.ncbi.nlm.nih.gov/Blast.cgi?CMD=Web&PAGE_TYPE=BlastDocs&DOC_TYPE=Download) and the validated and reviewed protein entries in RefSeq release 60 [36] (http://www.ncbi.nlm.nih.gov/refseq). They were also subjected to BLASTn v.2.2.29 analysis using *Medicago trancatula* Mt4.0 (http://www.medicagogenome.org) coding sequence (CDS) to extract their orthologues in the *M*. *trancatula* genome. The gene ontology (GO) terms were then assigned to up-regulated genes using Blast2Go software (http://www.blast2go.com/). Candidate defense response genes were selected from the list of up-regulated genes based on GO terms and published genes associated with defense pathways in plants. The expression levels of each of the candidate genes were compared among genotypes, and genes with difference in fold changes of greater than 2 units between at least two genotypes were considered differentially expressed among genotypes.

For genes annotated as resistance gene analogues (RGA), the DNA sequences of their conserved domains including nucleotide binding sites (NBS), leucine rich repeat (LRR), toll/interleukin 1 receptor (TIR) and coiled coil were identified by NCBI BlastX tool (https://blast.ncbi.nlm.nih.gov/Blast.cgi?LINK_LOC=blasthome&PAGE_TYPE=BlastSearch&PROGRAM=blastx). Genetic variants among genotypes within these domains and other coding regions of RGA genes were called by SAMtools v. 1.3.1 (https://academic.oup.com/bioinformatics/article/25/16/2078/204688) and filtered by VCFtools 1.13 (https://academic.oup.com/bioinformatics/article/27/15/2156/402296) for a minimum variant quality of 30, minimum SNP depth of 5 and minimum insertion/delition depth of 10. For genes annotated as mitogen activated proein kinases (MAPK), the CDC Redberry sequence was blasted against the chickpea and *M*. *trancatula* MAPK sequences previously reported by Purayannur [37] using standalone BLASTn v. 2.2.29 (https://blast.ncbi.nlm.nih.gov/Blast.cgi?CMD=Web&PAGE_TYPE=BlastDocs&DOC_TYPE=Download).

### Assessing the expression of selected candidate defense gene by quantitative real time-PCR (RT-qPCR)

To further confirm RNA-seq results for differential gene expression, the expression of a set of candidate genes was assessed using qRT-PCR. Samples used for qRT-PCR were the single biological replicate used for RNA-seq analysis plus two additional biological replicates of the same experiment described above. Primers were designed using lentil cv. CDC Redberry gene sequences with Primer3 software (http://biotools.umassmed.edu/bioapps/primer3_www.cgiwith) with the default parameters except for the maximum product size which was adjusted to 200 bp (Table 1). Total RNA (~1 μg) was used for reverse transcriptase-dependent first strand cDNA synthesis using the High-Capacity RNA to cDNA Kit^™^ (Applied Biosystems, Warrington, UK) following the manufacturer’s protocol. PCR amplifications were conducted in a CFX384 C1000 Touch^®^ real time thermo-cycler (Bio-Rad Laboratories Inc., Singapore) in a 12.5 μl reaction containing 7.1 μl of Applied Biosystems^®^ Fast SYBR^®^ Green Master Mix (Applied Biosystems, Warrington, UK), 0.2 μM of each primer and 5 μl of 1:10 diluted cDNA. The amplification conditions were 95 °C for 3 min, 40 cycles of 95 °C for 10 s, 60 °C for 30 s followed by a melting curve from 60 °C to 95 °C with 0.3 °C intervals. PCR reactions were conducted in triplicate and repeated if the standard deviation of the replicates was higher than 0.2.

**Table 1:** Primer pairs used for assessing the expression of selected candidate defense genes by quantitative real time PCR. Primers were designed using lentil cv. CDC Redberry sequences. *ABI1-B = Abscisic acid insensitive 1b; DDB1-CUl4 = ddb1- and cul4-associated factor; PRH = pathogenesis-related homeodomain; Pti1 = Pseudomonas syringae pv*. *tomato R-gene (Pto)-interactor 1*; *RGA1 and 71* = *resistance gene analogue 1 and 71*. Gene IDs were generated using Cufflinks software and link data presented here to the transcript annotations in S1 File.

Gene ID	Gene name	Sequence 5’->3’
TSS6729	*ABI1-B*	F: ATCCGAGGTACAATCGCAAC
		R: CCTTGGAAACGAAACAGGAG
TSS4370	*DDB1-CUL4*	F: CTCATCCACAGGGAACAAAAA
		R: GATTAGGTGACGAGGGCAAA
TSS7406	*PRH*	F: TCATCTGAGGGCCATTCTTC
		R: CATTCCTCCTGGAGACCAAG
TSS894	*Pti1*	F: GAGTTAAAATCGCCGTTGGA
		R: TCCAAGAACACGGGTAGAATG
TSS25883	*RGA1*	F:AGGAAAGAACGCTTGACTGG
		R: ACGGCTAGTAGCTGGGAATG
TSS15293	*RGA71*	F: ACCCAACGATTTTGATCAGG
		R: ATCTCCAATGGACGGGTGTA

Presense of residual genomic DNA contamination of total RNA samples was detected by running a PCR using ubiquitous actin primer pairs designed for an exon-exon junction and first strand cDNA following the protocol of Vaghefi et al. [38]. Amplification efficiency was calculated for each primer pair and lentil genotype using cDNA stock serially diluted 1:4 (V/V) five times for a total of 6 dilutions. Dilutions were used for qRT-PCR following the protocol described above. A linear equation was fitted to the cycle of threshold (Ct) values obtained for various cDNA dilutions. Percentile of amplification efficiency (E) was calculated from the slope of the regression line using the equation E = 10 ^(-1/slope)^ -1. New primer pairs were designed if E was lower than 99%.

QRT-PCR data were normalized using β-*actin* gene expression as a reference gene. The LcActin- 257 primer pairs [29] were used for amplification of β-*actin*. Expression level was reported relative to the mock-inoculated control by calculating fold changes in expression levels following the method of Livak and Schmittgen [39]. Normalized data were subjected to generalized linear mixed model analysis using the Statistical Analysis System (SAS) version 9.3 (SAS Institute Inc., Cary, NC, USA). Genotypes and sampling time points were considered fixed effects, replicates were random effects and sampling time points were identified as repeated measurements. A log-normal distribution with an identity link function was specified to account for the non-normal distribution, and a first-order antedependence covariance structure was used to accommodate unequally spaced sampling time points and heterogeneous variances. Differences among genotypes and sampling time points were assessed based on least significant differences with the Tukey adjustment (α = 0.05) in the generalized linear mixed model procedure. Spearman’s correlation analysis was conducted between expression fold change data obtained from RNA-seq and qRT-PCR analysis for the biological replicate used for RNA-seq analysis using PROC CORR of SAS.

## Results and discussion

### Gene expression profile of samples used for RNA-seq analysis

**Fig 1:**
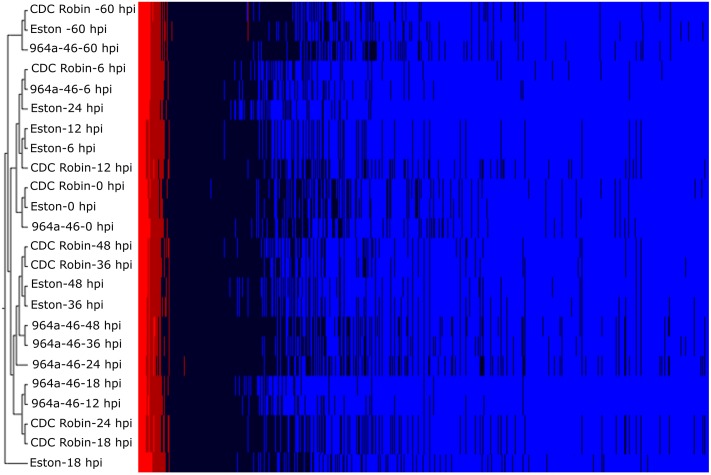
Hierarchical cluster analysis of gene expression profiles of lentil genotypes Eston, CDC Robin and 964a-46 measured at 0 (mock-inoculated control plants sampled before inoculation), 6, 12, 18, 24, 36, 48 and 60 hours post inoculation (hpi) with *Ascochyta lentis*. Heat map shows the normalized expression levels of transcripts represented by a color spectrum ranging from red (high expression levels) to blue (low expression levels). The dendrogram shows Pearson’s correlation with an average linkage distance among samples.

Samples used for RNA-seq analysis were clustered based on the expression of the entire transcriptome revealing three main groups with considerable variation in each group (Fig 1). The three genotypes had similar expression profiles at 0 hpi enabling comparative analysis among genotypes. When samples of each genotype were compared at different time points, the expression profile of Eston at 6, 12 and 24 hpi was similar to that at 0 hpi, whereas in 964a-46, expression at 0 hpi was similar to that at 6 hpi, and gene expression in CDC Robin at 0 hpi was similar to those at 6 and 12 hpi. This suggests that a delayed response in Eston may contribute to susceptibility. This agrees with previous studies suggesting that susceptible lentil genotypes display a delayed response to *A*. *lentis* infection compared to resistant genotypes [26,40]. The expression profile of 964a-46 was different from CDC Robin at 12 and 24 hpi.. At 12 to 18 hpi, the expression profile of 964a-46 was similar to that of CDC Robin at 18 to 24 hpi, supporting earlier activation of defense responses in 964a-46 than CDC Robin. A divergent gene expression pattern among genotypes was apparent mostly at 12, 18 and 24 hpi, indicating that most genes differentially expressed among genotypes were induced within 12 to 24 hpi. This is in agreement with the results of Khorramdelazad et al. [26] that most lentil defense responses to *A*. *lentis* infection occur prior to 24 hpi.

### Variation among genotypes in number of differentially expressed genes

**Fig 2:**
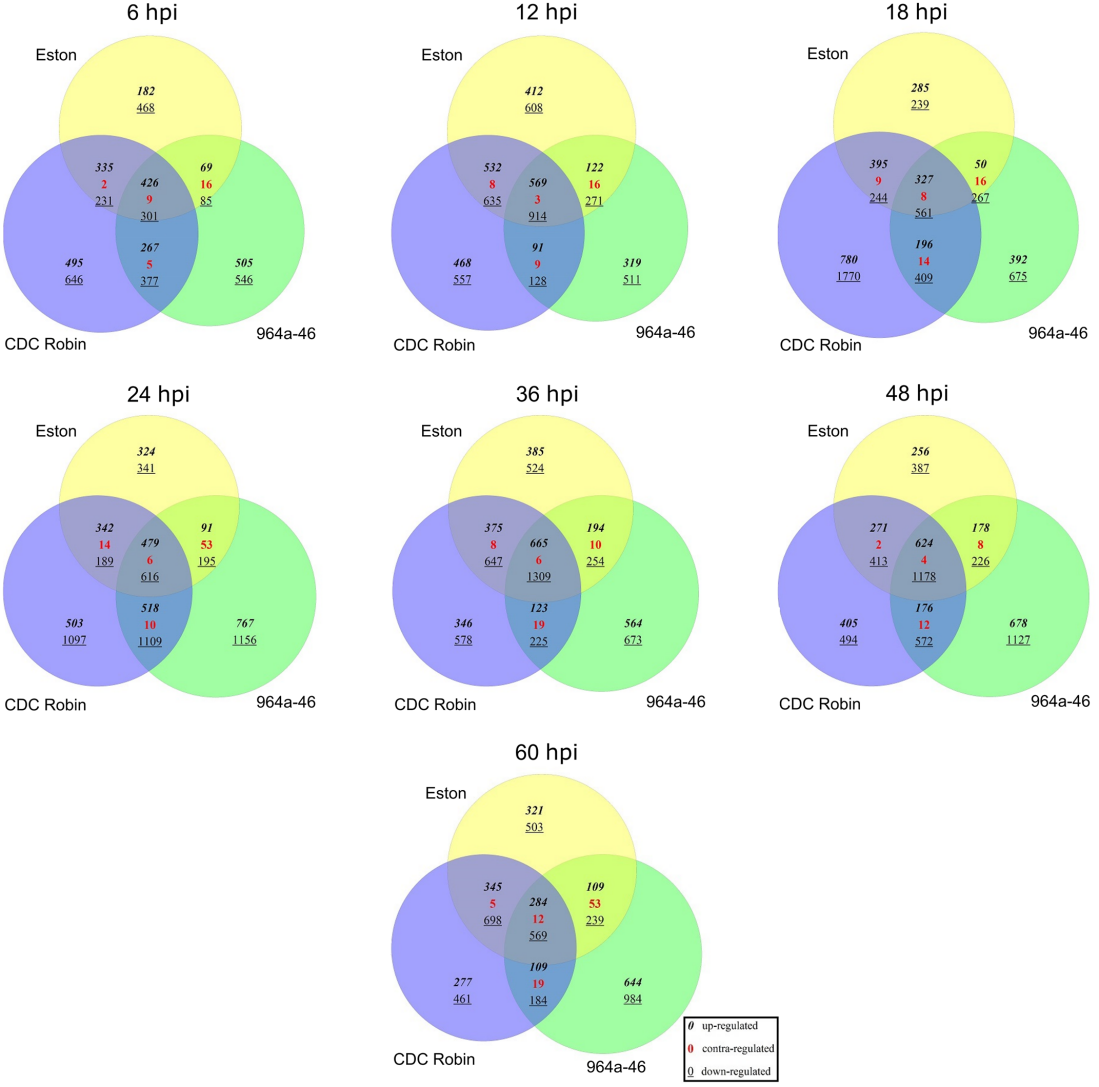
The number of unique and common differentially expressed genes among lentil genotypes Eston, CDC Robin and 964a-46 upon *A*. *lentis* infection. Hpi, hours post inoculation. A relative expression fold change of two compared with the mock inoculated plants samples at time 0 was considered as a threshold for determining the differentially expressed genes. Contra-regulated genes had contrasting expression levels in different genotypes.

The number of up-regulated genes was the highest at 12 hpi in Eston (2439 genes) but at 24 hpi it was higher in CDC Robin (1861 genes) and 964a-46 (1870 genes, S3 Table). The number of uniquely up-regulated genes in 964a-46 was several times higher than those commonly up-regulated between 964a-46 and Eston, but not in CDC Robin, at 6, 18 and 24 hpi (Fig 2), suggesting that Eston and 964a-46 deploy diverse transcripts in response to *A*. *lentis* at these sampling time points. Eston and CDC Robin had relatively lower numbers of unique up-regulated transcripts than 964a-46 at all time points except for 12 and 18 hpi, at which time points CDC Robin had more unique up-regulated transcripts than the other two genotypes. The same pattern was observed for down-regulated genes. The number of uniquely down-regulated genes in 964a-46 was higher than that of commonly down-regulated between Eston and 964a-46, but not in CDC Robin. The number of uniquely down-regulated genes in CDC Robin was seven times higher at 18 hpi and five times higher at 24 hpi compared to commonly down-regulated genes between Eston and CDC Robin (but excluding 964a-46), whereas these numbers were similar or lower at other time points. The number of contra-regulated genes (with contrasting expression levels) was often higher between Eston and 964a-46 than between Eston and CDC Robin, further supporting the divergence of defense responses deployed by Eston and 964a-46 in response to *A*. *lentis* invasion.

### Ontology enrichment analysis of genes up-regulated by *A*. *lentis* infection

**Fig 3:**
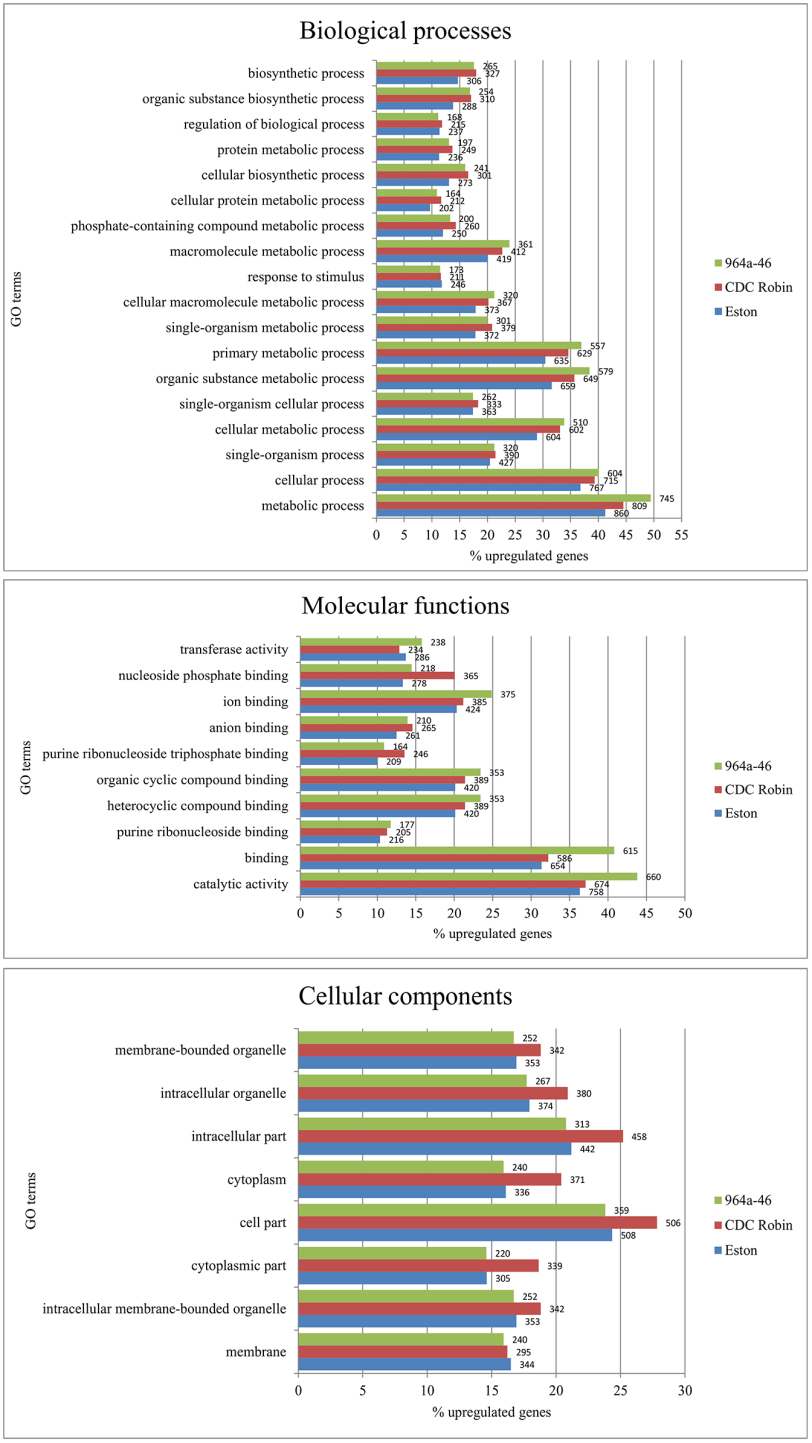
Gene ontology (GO) terms assigned to genes up-regulated in lentil genotypes CDC Robin, 964a-46 and Eston by *Ascochyta lentis* infection. GO enrichment analysis was conducted using Blast2Go software and the percentage of up-regulated genes belonging to each GO term was extracted from the tabular output of the combined graph analysis. Values associated with bars are the numbers of up-regulated genes of each GO term.

To obtain a general overview of what major biological, functional and cellular processes are mediated by the up-regulated genes and how the lentil genotype differed in these processes, the up-regulated genes were subjected to GO enrichment analysis. Results suggest that the majority of up-regulated genes had a role in “biological metabolite processes” in all three genotypes (Fig 3). Among the GO term categories associated with biological processes, the greatest difference between resistant genotypes CDC Robin and 964a-46 was in the “metabolite process” GO category which by definition includes genes involved in the transfer of small molecules, DNA repair and, protein synthesis and degradation. Previous results indicated that the majority of genes induced in *Nicotiana benthamiana* by *Verticillium dahliae* infection belonged to this GO category [41].

In the molecular function category, up-regulated genes belonged to “catalytic activity”, “transferase activity” and several types of “binding activity” GO terms. The GO term “catalytic activity” had the highest percentage of up-regulated genes among all GO terms of this category, with 964a-46 having the highest percentage of up-regulated genes among lentil genotypes. Resistant genotypes CDC Robin and 964a-46 had higher percentages of up-regulated genes in all sub-categories of the molecular function category compared to the susceptible check Eston, except for “transcriptase activity” for which CDC Robin had a slightly lower percentage than the susceptible check Eston. This may suggest a relatively higher ability of the resistant genotypes to induce defense responses belonging to various molecular functions and biological processes, resulting in their quicker response to infection.

Lines 964a-46 and Eston had similar percentages of up-regulated genes in all sub-categories of cellular components. The greatest difference between Eston and 964a-46, and CDC Robin was observed in the genes with “cytoplasm activity” GO terms with CDC Robin expressing more cytoplasmic genes than the other genotypesThis may suggest that cytoplasmic cellular defense mechanisms are more engaged in the reaction of CDC Robin to *A*. *lentis* than in the other genotypes.

### Defense response genes differentially expressed among lentil genotypes infected with *A*. *lentis*

Genes differentially expressed among genotypes and their transcripts along with their orthologues in *M*. *trancatula* are presented in S1 File. A number of genes tentatively involved in PTI including receptors and signaling components were differentially expressed among the genotypes. PTI-associated genes exclusively induced in Eston included *BAK1* and *cyclin dependent kinase* (*CDK*) (Table 2). *BAK1*-deficient *Arabidopsis* plants lost their ability to contain programmed cell death and as a consequence were more susceptible to necrotrophic pathogens [42]. Cheng et al. [43] suggested that brassinosteroid signaling mediated through *BAK1* and *Brassinosteroids Insensitive 1* (*BRI1*) interaction is similar in *Arabidopsis* and the model legume *M*. *trancatula*, supporting further the possible involvement of *BAK1* in perceiving *A*. *lentis* invasion in lentil. *BAK1* expression peaked at 6 hpi in Eston and declined at 12 hpi (data not shown). This decline could be due to the suppression of PTI by pathogen effectors. *NIMA-related kinase 6* (*NEK6*), *Receptor like kinase 1* (*RLK1*), *lectin s-receptor-like serine threonine-protein kinase* (*LERCK*) and *wall associated kinase-like protein* (*WAK*) are RLKs up-regulated exclusively in CDC Robin. *NEK6* encodes a serine/threonine protein kinase previously shown to be induced by 1-aminocyclopropane-1-carboxylic acid (ACC), the precursor of ethylene that is involved in response to stresses in *A*. *thaliana* [44]. Orthologues of *WAK* have also been characterized as PAMP receptors perceiving pectin monomers released during cell wall degradation by the pathogen’s pectinolytic enzymes [45]. *LERCK* is a RLK with a trans-membrane lectin domain that was initially characterized in legumes. It is required for β-aminobutyric acid (BABA)-mediated priming of resistance in *A*. *thaliana* [46]. Khorramdelazad et al. [26] recently reported temporal differences in the expression of a receptor like kinase in lentil genotypes ILL 7537 and ILL 6002, suggesting that its higher and earlier induction was associated with resistance. RLK are generally reported to have a positive role in resistance against necrotrophs e.g. ERECTA receptor like kinase mediates resistance to the necrotroph *Plectosphaerella cucumerina* in *A*. *thaliana* [47]. Some RLK serve as plant R genes e.g. products of *PBS1* [48] and are involved in the specific recognition of pathogen avirulence gene (*avr*) products. An orthologue of *Pseudomonas syringae* pv. *tomato* R-gene *(Pto)-interactor 1* (*Pti1*) was among the genes up-regulated only in Eston. This gene encodes a protein kinase involved in the signaling downstream of specific recognition of AvrPto by Pto [43]. This indicates the presence of AvrPto/Pto-like recognition system that could induce cell death in Eston, probably rendering Eston susceptible to ascochyta blight. The role of RLK in resistance to ascochyta blight needs to be evaluated in future studies.

**Table 2:** Sequence description and expression levels of candidate plant defense genes differentially expressed among lentil genotypes infected with *Ascochyta lentis*. Sequence descriptions are from the BLASTx against RefSeq release 60 hits with the highest percentage of sequence identity. Gene symbols were extracted from the *Arabidopsis* information resource TAIR (http://www.arabidopsis.org). For genes with no gene symbol in TAIR, the abbreviation of sequence description was used. Fold change in gene expression was calculated by Cuffdiff software by dividing fragments per kb of exon per million mapped reads (FPKM) value of infected samples by that of non-infected sample collected before inoculation (mock). NA = A copy of PR-4a gene was absent in 964a-46. Hpi = hours post *Ascochyta lentis* inoculation. Gene IDs were generated using Cufflinks software and links data presented here to the transcript annotations in S1 File.

Gene ID	Sequence description	Gene symbol	Genotypes					
			Eston		CDC Robin		964a-46	
			Peak time (hpi)	Log_2_ fold change	Peak time (hpi)	Log_2_ fold change	Peak time (hpi)	Log_2_ fold change
TSS653	serine/threonine-kinase Nek6-like protein	*NEK6*	12	1.0	12	15.0	36	1.0
TSS25223	receptor-like protein kinase 1	*RLK1*	48	2.0	24	14.9	60	0.9
TSS20893	wall associated kinase-like protein	*WAK*	18	6.0	6	15.2	12	4.5
TSS894	Pto kinase interactor 1	*Pti1*	36	15.7	24	2.3	48	-0.2
TSS13714	S-locus lectin kinase family protein	*LECRK*	12	4.6	48	13.6	18	-0.1
TSS15246	leu-rich receptor serine threonine protein kinase bak1	*BAK1*	6	11.8	12	1.9	6	1.0
TSS25469	efr3-like protein	*EFR3*	6	15.2	36	1.2	24	0.8
TSS15452	cbl-interacting protein kinase	*CIPK*	48	12.1	24	3.0	6	14.1
TSS20721	mitogen-activated protein kinase 3 (ERK1)	*MAPK3*	12	12.2	6	-0.5	6	-0.3
TSS16912	mitogen activated protein kinase 20–1	*MAPK 20–1*	36	3.4	18	14.3	12	11.2
TSS12352	kinase-like protein WNK11	*WNK11*	36	1.3	24	11.6	24	1.1
TSS21203	map kinase homolog ntf6-like	*MAPK-ntf6*	24	0.5	36	1.0	36	12.9
TSS24130	mitogen-activated protein kinase kinase	*MAPKK*	12	2.0	6	2.3	18	12.5
TSS11951	MAPK/erk kinase 2	*MEK2*	18	0.2	24	13.4	6	1.1
TSS3202	ET-responsive transcription factor 1b-like	*ERF1b*	12	3.4	6	1.5	6	17.4
TSS13281	ET-responsive transcription factor wri1	*WRI1*	36	12.6	18	14.2	6	1.7
TSS20180	ET-responsive transcription factor	*ERF*	18	0.5	36	1.1	36	11.0
TSS17746	ethylene receptor-like	*ER*	36	1.7	24	9.7	6	10.8
TSS12237	gaga-binding transcriptional activator	*GAGA-TF*	36	0.3	12	0.1	12	11.3
TSS6729	abscisic insensitive 1b	*ABI1-B*	24	0.9	18	11.2	36	-0.7
TSS9333	abscisic acid-insensitive 5-like protein	*ABI5*	6	14.7	60	0.1	60	0.9
TSS10835	pentatricopeptide repeat-containing protein	*PRP*	36	-0.4	36	10.7	24	2.1
TSS17847	f-box protein skip16	*SKIP16*	18	0.3	12	9.9	12	0.2
TSS22596	f-box fbd lrr-repeat protein at3g14710-like	*FBD*	60	3.4	60	4.8	60	18.2
TSS14333	f-box lrr-repeat protein	*FLR*	60	13.7	18	0.7	36	2.7
TSS4370	ddb1- and cul4-associated factor	*DDB1-CUL4*	48	2.5	24	-0.7	48	12.0
TSS4644	ankyrin repeat domain-containing protein	*AKR*	12	-0.6	12	12.3	18	-0.2
TSS4558	pathogenesis-related protein 1a	*PR-1a*	18	6.8	12	3.4	36	11.3
TSS19333	Heat shock protein	*HSP*	12	13.5	18	1.3	24	11.6
TSS4129	Hevein-like protein	*Hel*	60	1.9	12	8.0	12	2.0
TSS3616	pathogenesis-related protein pr-4a- (Copy#1)	*PR-4a*	12	2.7	18	8.6	24	9.4
TSS27157	pathogenesis-related protein pr-4a (Copy#2)	*PR-4a*	12	2.7	18	7.8	NA	NA
TSS7944	thaumatin-like protein	*TLP*	12	1.7	12	5.9	24	6.0
TSS16562	calcium-transporting atpase	*CTA*	12	0.6	12	0.8	36	13.3
TSS10753	programmed-cell death protein-1	*PDCD-1*	60	10.1	60	-0.3	18	11.1
TSS25553	cyclin-dependent kinase g-2-like	*CDK*	36	11.4	24	0.7	24	0.4
TSS12093	autophagy-related protein	*ATG*	6	13.6	12	12.6	6	1.1
TSS5841	autophagy-related protein 18g-like	*ATG18g*	18	1.9	6	14.2	36	1.8
TSS18849	cellulose synthase h1-like	*CESA*	12	1.7	6	9.7	24	0.7
TSS16022	xyloglucan glycosyltransferase 6-like	*CSLC6*	6	6.4	18	14.4	12	10.4
TSS25181	callose synthase 11-like isoform x1	*CALS*	36	2.0	18	1.1	36	11.4
TSS7406	pathogenesis-related homeodomain	*PRH*	12	0.7	24	1.9	36	16.2
TSS948	tga transcription factor	*TGA*	6	7.9	12	1.9	48	-0.3
TSS23398	myb-like dna-binding protein bas1	*MYB*	24	11.0	6	2.9	36	10.3
TSS4173	poly polymerase-like	*PARP*	48	5.7	48	12.6	6	0.7
TSS6941	arginine amidohydrolase	*ARGAH*	48	3.6	12	7.2	36	1.9

**Fig 4:**
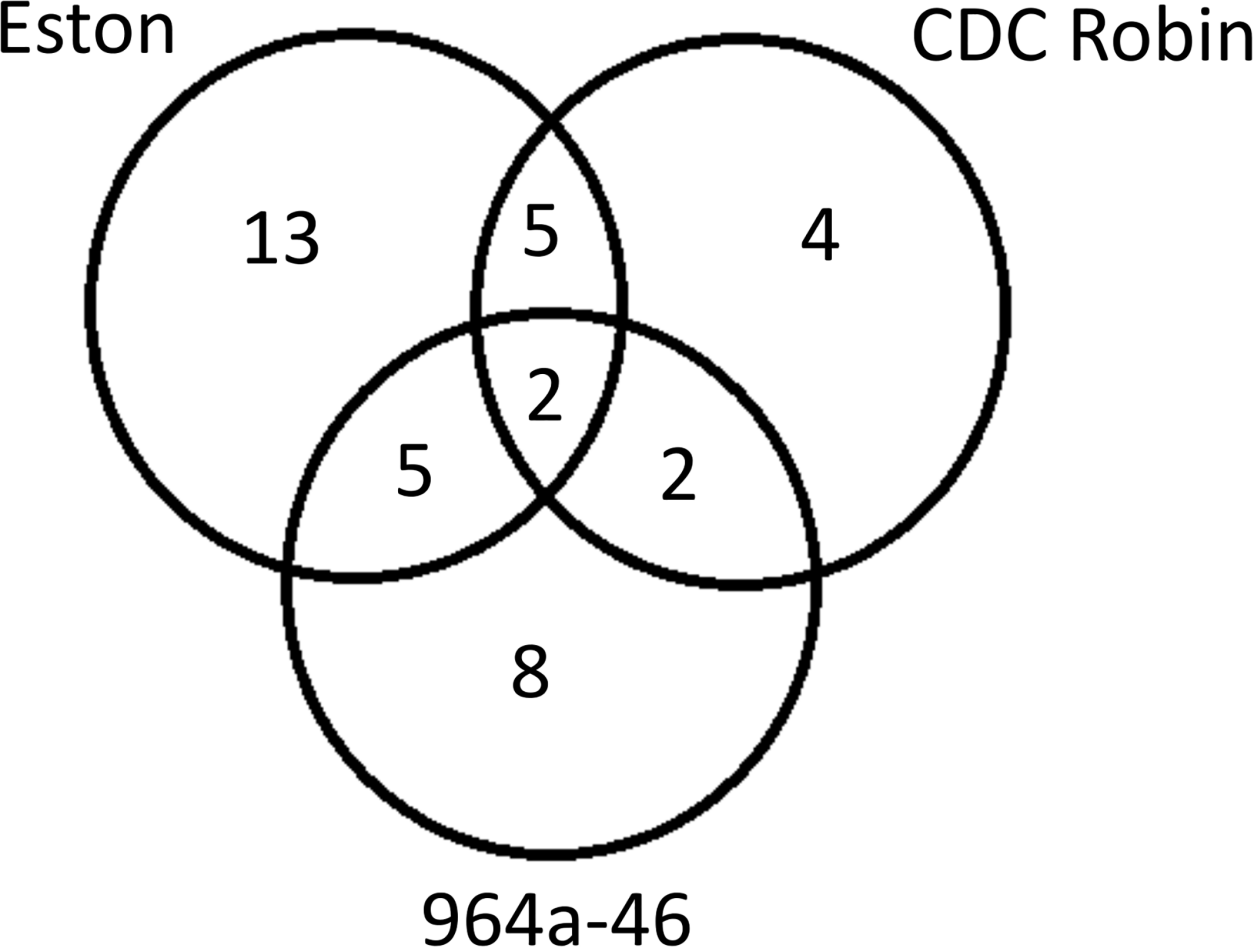
Venn diagram showing the number of nucleotide binding site-leucine rich repeat (NBS-LRR) resistance genes differentially expressed among lentil genotypes CDC Robin, 964a-46 and Eston after *Ascochyta lentis* infection.

NBS-LRR genes are involved in the second layer of pathogen recognition and perceive pathogen effectors that suppress PTI [49]. A total of 32 NBS-LRR genes were differentially expressed in response to *A*. *lentis* infection (S4 Table), among which 13 were exclusively up-regulated in Eston whereas the up-regulation of 12 was similar between Eston and the other two genotypes (Fig 4). Only two NBS-LRR genes were up-regulated in all three genotypes, indicating distinction among genotypes in deploying NBS-LRR genes for the perception of *A*. *lentis* effectors. In addition to differences in the number of up-regulated NBS-LRR genes among genotypes, those up-regulated in all genotypes also differed in the peak time and expression levels. Due to the importance of NBS-LRR genes in plant defense, the genetic variants associated with them and their association with conserved domains of the NBS-LRR genes were reported (S2 File). Genetic variants of large impact that interfered with the transcription of genes were observed in *RGA1*, *RGA12*, *RGA17*, *RGA37*, *RGA41* and *RGA49* (S2 File). 964a-64 had no reads mapped to *RGA12*, *RGA17* and *RGA37* loci, inferring that 964a-46 either carries a deletion mutation or a large impact mutations at upstream regulatory elements that interfere with the transcription of these genes. This might explain why *RGA12* and *RGA17* were exclusively expressed in Eston, whereas *RGA37* had contrasting expression levels in Eston and 964a-46. Transcripts for *RGA1* and *RGA49* appear to be truncated in 964a-46 and those for *RGA41* in Eston considering that no reads were mapped to a portion of these genes in samples of these genotypes (S2 File). The fact that these genes were not fully transcribed in 964a-46 and Eston further supports the presence of two distinct strategies for resistance in 964–46 and CDC Robin, which was also observed in the whole transcriptome comparision of these genotypes (Fig 2). *N* encodes a TIR-NBS-LRR gene, mediating resistance to Tobacco Mosaic Virus (TMV) through the induction of HR [50]. The up-regulation of *N* orthologues in Eston and 964a-46, both of which show cell death upon *A*. *lentis* infection [29], supports the hypothesis that these genes are hijacked by *A*. *lentis* for the induction of cell death. All together, these findings indicated that NBS-LRR genes form a dominant family of genes differentially expressed among the lentil genotypes upon *A*. *lentis* infection. This agrees with Garcia et al. [25] reporting that 31 NBS-LRR genes were up-regulated in lentil genotype ILL 5588 after *A*. *lentis* infection, emphasizing further the involvement of these genes as a response to *A*. *lentis* infection.

Except for RLM3, a TIR-NBS-LRR protein that mediates resistance to the hemibiotroph *L*. *maculans* and the necrotrophs *B*. *cinerea*, *Alternaria brassica* and *A*. *brassicicola* in *A*. *thaliana* [51], none of the NBS-LRR genes characterized to date have been associated with resistance to necrotrophs. The involvement of NBS-LRR genes in containing the invasion of *A*. *lentis* through induction of HR was proposed in a transcriptome analysis of LL 7537 genotype [26], however no ETI has been thus far reported for the lentil-*A lentis* interaction. NBS-LRR genes mediate susceptibility to host-specific necrotrophs such as *Pyrenophora tritici-repentis* and *S*. *nodorum* in wheat through interaction with the host-specific effectors (toxins) [52]. The up-regulation of a higher number of NBS-LRR genes in the susceptible check Eston may support the involvement of a complex toxin model in the interaction of lentil and *A*. *lentis*. Further genetic studies are needed to reveal the role of NBS-LRR genes in the interaction of lentil to *A*. *lentis*. Regardless of the positive or negative contribution of NBS-LRR genes to ascochyta blight resistance in lentil, identification of differentially expressed NBS-LRR in this and previous studies will be instrumental for dissecting major ascochyta blight resistance genes in lentil. This could be facilitated through comparative mapping analysis to identify NBS-LRR genes co-located with major QTL identified for these genotypes [53], but will also require functional characterization of these genes.

Mitogen activated protein kinase (MAPK) signaling is a common process in eukaryotes and is involved in transducing signals of abiotic and biotic stresses in plants [54]. This pathway is involved in the transduction of signals generated from both NBS-LRR and PRR, inducing the expression of many downstream defense pathways involved in plant hormone synthesis, generation of ROS, induction of PR proteins and cell death [54]. However, MAPKs are targeted by some pathogen effectors to subvert PTI and ETI [55,56]. Several orthologues of MAPK genes including *MAPK3*, *MAPK20-1*, *MAPK-ntf6*, *MAPKK* and *MEK2* were differentially expressed among genotypes here. Except for *MAPK3*, they were up-regulated in either one or both resistant genotypes, supporting an association between MAPK activities and the resistance to ascochyta blight in lentil. *MAPK/erk kinase 2* (*MEK2*) was only up-regulated in CDC Robin while *MAPK-ntf6* and *MAPKK* in 964a-46. *MEK2* has a function in protein phosphorylation during PAMP signaling and interacts with *SNF-related kinase* (*SNRK*) which was induced in all genotypes in the present study (S5 Table). Recently, MAPK signaling was characterized in multiple legume species [37]. Comparing the CDC Redberry sequences with the MAPK genes reported by Purayannur et al. [37] in chickpea (*Cicer oreintanum*) and *M*. *trancatula* showed 92.6% sequence identity between *MAPK20-1* (LC34453) and *CaMAPK3* (LOC101510662), 93.5% between *MAPK-ntf6* (LC19505) and Medtr4g061320, 87.2% between *MAPKK* (LC08191) and Medtr4g005830 and 93.4% between *MEK2* (LC14914) and *CaMAPK7* (LOC101489531). This indicates that MAPK signaling pathway is highly conserved among different legumes species. Purayannur et al. [37] reported a direct interaction between *CaMAPK3 and CaMAPK7*, indicating the potential for a direct interaction between *MEK2* and *MAPK20-1* in the present study. The up-regulation of *MEK2* and *MAPK20*-1 in CDC Robin further supports that these genes function together in MAPK signaling in this genotype. It is likely that the signal transduced by *MEK2/MAPK20-1* is interrupted in 964a-46 since *MAPK20-1*, but not *MEK2*, was induced in this genotype. The role of *MEK2/MAPK20-1* in the resistance of CDC Robin to ascochyta blight needs to be evaluted in future studies.

A few differentially expressed genes were associated with protein-protein interaction contrbuting to downstream signal transduction pathways including *ankyrin repeat domain-containing* (*AKR)*, *ddb1- and cul4-associated factor* (*DDB1-CUL4*), and three genes encoding members of F-box proteins including *f-box protein skip16* (*SKIP16*), *f-box fbd lrr-repeat protein at3g14710-like* (*FBD*) and *f-box lrr-repeat protein* (*FLR*). An orthologue of *AKR* was up-regulated in CDC Robin but not in the other genotypes. *AKR* is a key part of hormonal signaling during plant defense [57,58]. Mustafa et al. [24] reported the suppression of an *AKR* in a lentil genotype susceptible to ascochyta blight, supporting a role of this gene in resistance. *DDB1-CUL4* and *FBD* were exclusively up-regulated in 964a-46, while *SKIP16* expression was specific to CDC Robin. *DDB1-CUL4*, *FBD* and *SKIP16* are involved in the regulation of various signaling pathways at translational levels. Interpreting the role of these genes in regulating ascochyta blight resistance requires further understanding of defense signaling pathways in lentil.

A few genes associated with the ABA signaling pathway were differentially expressed among genotypes. The expression of *abscisic insensitive 1b* (*ABI1-B*) which is a negative regulator of ABA signaling [59] was only induced in CDC Robin. This could indicate a potential role of ABA suppression on resistance to ascochyta blight in CDC Robin. *ABI1-B* encodes a serine/threonine protein phosphatase 2C that lowers responsiveness to ABA in plants [59]. A negative regulation of ABA signaling by *ABI1* was previously reported for *M*. *trancatula* [60], suggesting that *ABI1* function is conserved among different plant species. *Pentatricopeptide repeat-containing* (*PRP*) which is an orthologue of *ABA overly-sensitive 5* (*ABO5*) in *A*. *thaliana* was exclusively induced in CDC Robin. The regulation of the ABA signaling pathway was previously suggested for this gene [61]. Meanwhile, the induction of *abscisic insensitive* 5 (*ABI5*) that mediates hypersensitivity to ABA [62] in Eston suggested the induction of the ABA pathway in this genotype. Previous studies indicated an antagonistic effect of ABA on the SA and JA/ET signaling pathways [63,64]. For example, ABA made *A*. *thaliana* more susceptible to *F*. *oxysporum* through suppressing the JA/ET-induced defense genes such as *plant defensin like protein 1*.*2* (*PDF1*.*2)* and *PR-4a* [63]. A similar mechanism could be postulated in the susceptible genotype Eston as the expression of *PR-4a* was much lower in this than in the partially resistant genotypes CDC Robin and 964a-46 as shown previously [29], and confirmed again here.

Previously, Sari et al. [29] showed that *PR-1a* expression that was initially induced in Eston early during infection by *A*. *lentis* and down-regulated thereafter, had two peak at 24 and 48 hpi in 964a-46 [29]. This may further support the hypothesis of suppression of the SA signaling pathway in Eston. *PR-1a* was induced in Eston here as well, and at lower expression levels than in 964a-46 and CDC Robin. In the previous study [29], the induction of SA could be associated with the lower *A*. *lentis* colonization observed for 964a-46 indicating a possible role in resistance. In support of this, Ferrari et al. [16] proposed a role for the SA signaling pathway in resistance to *B*. *cinerea*. However, the SA pathway is generally involved in the induction of resistance against biotrophs, and induced during the biotrophic phase in hemibiotrophs [65,66]. Other reports show that some necrotrophs hijack resistance mechanisms effective against biotrophs to induce cell death, which promotes host cell colonization by necrotrophs [67,68]. For example, transcriptome analysis of lettuce after *B*. *cinerea* infection revealed that similar defense pathways are activated in compatible reaction to this pathogen and incompatible reaction to *Bremia lactucae*, a biotroph causing powdery mildew in lettuce [69]. The SA pathway might be triggered by *A*. *lentis* effectors to promote cell death in 964a-46 and Eston. *DDB1-CUL4* that was induced at higher levels in 964a-46 than in both Eston and CDC Robin, is involved in ubiquitin-proteasome regulation of plant hormone signaling [70], and could also be associated with the strong activation of SA signaling in 964a-46. *PR-1a* and *DDB1-CUL4* expressions were lower in CDC Robin than in the other two genotypes supporting that SA signaling is either not triggered or suppressed during the defense responses to ascochyta blight in this genotype. The phytohormone signaling pathways are poorly understood in legumes, and it is possible that their activation and regulation in model plants such as *A*. *thaliana* which we used here to interpret our results, may vary from what takes place in lentil. Confirmation of genes involved in the SA signaling pathway in lentil and their role in resistance to ascochyta blight is required before the role of SA signaling in resistance can be determined conclusively.

*ET-responsive transcription factor wri1* (*WRI1*) was induced earlier and had higher expression levels in 964a-46 than in Eston. Three other ET-responsive transcription factors, *ethylene response factor 1b* (*ERF1b*), *ERF* and *GAGA-transcription factor* (*GAGA-TF*) were expressed exclusively in 964a-46. This suggested a higher participation of the ET signaling pathway in the interaction of 964a-46 with *A*. *lentis* than in CDC Robin, and could be the cause of the lower colonization of 964a-46 by *A*. *lentis* than Eston. Khorramdelazad et al. [26] reported a ET response factor (ERF) gene that had higher expression at the earlier phase of infection in ILL 7537 but at later phase of infection in ILL 6002 genotypes. Most of the ET/JA-associated genes detected in the present study have not been characterized in lentil to date, therefore their role in plant defense need to be studied in future.

An orthologue of *Programmed-cell death-1* (*PDCD-1*) was up-regulated in Eston and 964a-46, but not in CDC Robin. The expression of this gene along with *CDK* in Eston might have contributed to the induction of cell death by *A*. *lentis* infection in this genotype. The expression of *PDCD-1* peaked earlier in 964a-46 than in Eston. The absence of *CDK* induction in 964a-46 and the difference between Eston and 964a-46 in the timing of *PDCD-1* induction supports the differences in the manifestation of cell death between Eston and 964a-46. *Autophagy-related 18g-like* (*ATG18g*) is involved in the inhibition of cell death through formation of autophagosomes [71]. This gene was induced only in CDC Robin and may contribute to cell death inhibition in this genotype. Sari et al. [29] previously suggested that cell death inhibition might be a mechanism of resistance to ascochyta blight in CDC Robin, which is supported by the expression of *ATG18g* here. A crucial role of autophagy in plant resistance to necrotrophs through negative regulation of cell death was previously suggested [71,72]. The inhibitory role of *ATG18g* is dependent on the JA signaling pathway and a WRKY transcription factor in *A*. *thaliana* [71], and the involvement of the JA signaling in resistance of CDC Robin to ascochyta blight has already been demonstrated [29]. Previous transcriptome analysis identified genes associated with cell death in ILL 7537; however, they contributed to resistance by promoting cell death and HR [26]. This could indicate differences in the response to *A*. *lentis* between CDC Robin and ILL 7537 considering that ILL 7537 responded to pathogen penetration by rapid generation of ROS and HR [40], whereas cell death inhibition contributed to resistance of CDC Robin [29]. From a breeding perspective, such different meachanisms are interesting as their combination may result in more durable resistance.

Genes involved in cell wall modification were differentially expressed among genotypes. *Poly polymerase-like* (*PARP*) was induced both in Eston and CDC Robin; however, the expression level was approximately two-times higher in CDC Robin at 48 hpi. *PARP* encodes a poly(ADP-Rib) polymerase enzyme that regulates various defense responses such as callose and lignin deposition, pigment accumulation, phenylalanine ammonia lyase activity and cell death inhibition [73]. A *PARP*-deficient *A*. *thaliana* mutant showed higher susceptibility to *B*. *cinerea*, suggesting the involvement of this gene in resistance to necrotrophs [73], hence higher *PARP* expression levels in CDC Robin may also be linked to higher resistance to ascochyta blight. *Xyloglucan glycosyltransferase 6-like* (*CSLC6*) is involved in the synthesis of xyloglucans that form the matrix polymers of the plant cell wall [74]. The higher expression of *CSLC6* in CDC Robin and 964a-46 compared to the susceptible check Eston correlates with resistance to ascochyta blight. *Cellulose synthase h1-like* (*CESA*) was only expressed in CDC Robin, while *callose synthase 11-like* (*CALS*) was expressed in 964a-46, suggesting different modes of cell wall alteration in these genotypes in response to *A*. *lentis* infection. Studies by Lorenzo et al. [75] indicated activation of *CESA* by ERF1, a transcription factor that integrates responses to the ET and JA signaling pathways. The negative regulation of ABA signaling in CDC Robin through up-regulation of genes such as *ABI1-B* could allow the induction of the JA signaling pathway, resulting in ERF1-mediated expression of *CESA* and thus limit colonization by *A*. *lentis* as observed in previous histopathological studies [29]. It is likely that the cell wall reinforcement mediated by the higher expression of *PARP*, *CSLC6* and *CESA* along with the negative regulation of cell-death contributed to delayed colonization of CDC Robin by *A*. *lentis* as suggested by Sari et al. [29]. It is also likely that the resistance of CDC Robin is associated with preformed physical barrier such as phenolic compounds deposited in the primary cell wall, reducing the penetration success of *A*. *lentis*. The genes associated with cell wall reinforcement induced in ILL 7537 [26] were different from those reported here, possibly because, unlike CDC Robin, ILL 7537 was unable to delay *A*. *lentis* colonization. The role of cell wall reinforcement and preformed physical barriers in resistance of CDC Robin requires further investigation.

Downstream defense response genes, mainly PR proteins, were also differentially expressed among genotypes. *PR-4a* and *PR-1a* were expressed differentially among genotypes. Reads associated with *PR-4a* were mapped to two contigs in genotype Eston and CDC Robin, but to only one in 964a-46. This could either indicate large variations between the sequences of CDC Redberry and 964a-46 at this locus, impeding the mapping of the read to the reference genome, or the presence of a deletion mutation of the second copy of *PR-4a* in 964a-46. Nevertheless, the expression of the *PR-4a* copy shared among genotypes was higher in resistant genotypes CDC Robin and 964a-46 than susceptible check Eston. *PR-4a* is the only PR protein studied in lentil to date. Previous studies suggested up-regulation of *PR-4a* upon *A*. *lentis* infection in resistant but not in susceptible genotypes [24,29]. Vaghefi et al. [38] demonstrated the antifungal activity *in vitro* of a recombinant lentil PR-4a protein (product of LcPR4a) on *A*. *lentis*. *Thaumatin-like protein* (*TLP*) was up-regulated in both resistant genotypes CDC Robin and 964a-46. *TLP*s belong to the PR-5 family which is a well-known SA-responsive group of genes [76]. However, some studies have suggested the expression of *TLP*s as an integrated response to both the SA and JA signaling pathways [77]. The fact that the SA signaling pathway was not activated in CDC Robin supports further the involvement of the JA signaling in the induction of *TLP*. Khorramdelazad et al. [26] also reported difference between ILL 7537 and ILL 6002 in induction of a PR4-thaumatin like protein, supporting that *TLP* are induced as a conserved defense response to *A*. *lentis* infection in various lentil genotypes. *Hevein-like* (*HEL*) was only up-regulated in CDC Robin. *HEL* is a JA-responsive genes used frequently as a marker for the JA signaling pathway and may contribute to the resistance of CDC Robin to ascochyta blight. Expression of this gene in CDC Robin confirmed the previous results on the involvement of the JA signaling in resistance of this genotype to ascochyta blight [29]. *Pathogenesis-related homeodomain* (*PRH*) was only induced in 964a-46. This gene encodes a transcription factor regulating the expression of PR proteins in response to pathogen attacks [78]. *Arginine amidohydrolase* (*ARGAH*) expression was higher in CDC Robin than the other genotypes. This gene is a key component of polyamine biosynthesis in plants and is involved in the defense responses induced by the JA signaling pathway in *A*. *thaliana* [79]. Knock-out and constitutive expression of *ARGAH* in *A*. *thaliana* has confirmed a positive role of this gene in resistance to *B*. *cinerea* [80].

### Assessing the expression levels of selected candidate genes by qRT-PCR

To further confirm RNA-seq results for differential gene expression, the expression of a set of candidate genes was assessed using qRT-PCR. Genes with different roles were selected for qRT-PCR analysis, including orthologues of *Pti1*, which plays a role in transducing signal downstream of Pto/AvrPto recognition, two resistance gene analogues *RGA1* and *RGA71* with putative roles as receptors, orthologues of *ABI1-B* and *DDB1-CUL4* with roles in signal transduction pathways and an orthologue of *PRH*, a transcription factor involved in the transcription activation of PR proteins.

**Fig 5:**
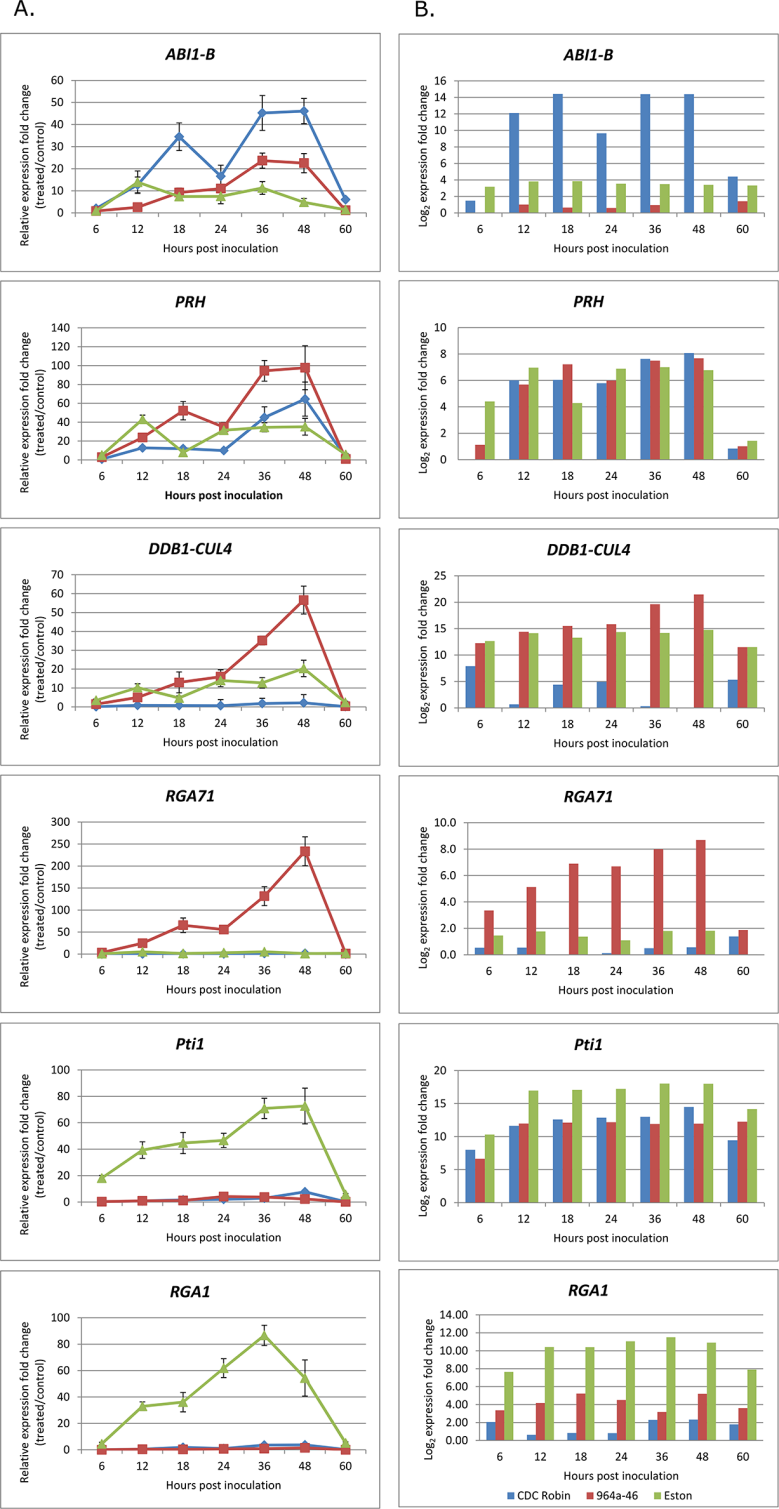
Differential expression of *Abscisic acid insensitive 1b* (*ABI1-B*), *ddb1- and cul4-associated factor* (*DDB1-CUl4*), *pathogenesis-related homeodomain* (*PRH*), *Pseudomonas syringae* pv. *tomato R-gene* (*Pto*)-*interactor 1* (*Pti1*), *resistance gene analogue 1* (*RGA1*) and *resistance gene analogues 71* (*RGA71*) among lentil genotypes infected with *Ascochyta lentis*. **A.** Expression data (means and standard deviations) measured by quantitative real-time PCR (qRT-PCR) based on three biological replicates. Cycle of threshold (C_t_) values were normalized with plant β-*actin* gene values as a reference gene and the gene expression was reported relative to non-infected plants sampled before inoculation (mock). **B.** RNA-seq data were generated from samples of one of the three biological replicates used for qRT-PCR. The log_2_ fold change in gene expression was calculated with Cuffdiff software by dividing fragments per kb of exon per million mapped reads (FPKM) value of infected samples to that of mock.

Based on RNA-seq analysis, the expression of *ABI1-B* was higher in CDC Robin, while that of *PRH*, *DDB1-CUL4* and *RGA71* was higher in 964a-46, and that of *Pti1* and *RGA1* was higher in Eston compared to the other genotypes (Fig 5A). Similar expression patterns were observed for qRT-PCR gene expression results and confirmed the differences among genotypes in the expression pattern for all six candidate defense genes (Fig 5B). For instance, *ABI1-B* expression declined in Eston at 18 hpi, while it reached a peak level in CDC Robin at this time. *DDB1-CUL4* and *PRH* expressions were significantly higher in 964a-46 than in the other two genotypes at 36–60 hpi (*DDB1-CUL4*), and 24 and 48 hpi (*PRH*). *PRH* expression was higher in Eston than 964a-46 at 12 hpi, however it declined at 18 hpi to a significantly lower level than that in 964a-46. *DDB1-CUL4* expression increased later than *ABI1-B* and *PRH* at 24 hpi and was significantly higher in 964a-46 than Eston at 36 and 48 hpi, and CDC Robin at 24–48 hpi. *Pti1* expression was significantly higher in Eston than in the two other genotypes at all sampling times, except for 60 hpi. *RGA1* and *RGA71* were highly expressed in Eston and 964a-46, respectively, which supported the results of RNA-seq analysis.

Correlations between the gene expression levels detected by RNA-seq and RT-qPCR were significant for all the six candidate genes tested using qRT-PCR (*P* < 0.001). The Spearman’s correlation coefficient between the RNA-seq and qRT-PCR fold changes values varied from 0.50 to 0.94, was highest for *PRH* and lowest for *ABI1-B*. This is similar to the results of De Cremer et al. [69], who used RNA-seq for dissecting the interaction of lettuce with *B*. *cinerea*. Li et al. [81] also evaluated the Spearmen’s correlation between the RNA-seq and qRT-PCR analysis of 996 genes and obtained correlation coefficients ranging from 0.56 to 0.68 when the FPKM method was used for normalization of RNA-seq data.

## Conclusions

Using the lentil reference genome [27] for RNA-seq analysis allowed a more comprehensive and less biased description of lentil defense transcriptome activated by *A*. *lentis* than that reported in previous study using *de novo* assembly of transcriptome [26]. The results revealed differences between ascochyta blight resistant genotypes CDC Robin and 964a-46, and the susceptible Eston for pathogen recognition, signal transduction pathways and the activation of downstream defense response genes. Previous reports on tomato and wheat indicated genotype-specific differences in the activation of resistance mechanisms, pathways and genes [82,83]. Such genotype-specific differences in components of defense mechanisms have been demonstrated for the first time here for ascochyta blight of lentil.

A large number of the genes differentially expressed among the lentil genotypes have functions downstream of pathogen recognition such as signaling pathways and defense response genes. Several other genes involved in pathogen recognition were also identified. These genes are of primary interest for mapping since they may be deployed for breeding of resistant cultivars. Genes differentially expressed among the genotypes and tentatively involved in the pathogen recognition were members of NBS-LRR and RLK gene families. The up-regulation of a large number of NBS-LRR genes in the genotype susceptible to *A*. *lentis* in the current study suggests that NBS-LRR genes may contribute to the susceptibility of lentil to ascochyta blight. It is conceivable that compatibility is the result of specific recognition of *A*. *lentis* host-selective effectors by NBS-LRR genes leading to the induction of cell death. The presence of genetic variants with large impact in some NBS-LRR genes differentially expressed among genotypes could be linked with the presence of non-allelic resistance genes in 964a-46 and CDC Robin. The lower colonization in 964a-46 compared to Eston may be associated with i) activation of the ET pathway early during infection, ii) transmission of a systemic signal to intact cells and priming resistance for subsequent infection, and iii) activation of the SA pathway and accumulation of large amounts of antimicrobial compounds which slowed down colonization.

Some of the well-known RLK, such as an orthologue of *BAK1*, were up-regulated in the susceptible genotype Eston. This was not surprising as many of the PTI responses are activated at the beginning of the infection process and subsequently suppressed by effectors of compatible pathogens. The present results show that different set of RLK were up-regulated in CDC Robin compared to susceptible Eston. Most of the resistance genes characterized for necrotrophs belong to RLK family. RLK up-regulated exclusively in CDC Robin serve as candidate resistance genes.

A complex signal transduction network emerged downstream of *A*. *lentis* recognition that involved the phytohormones SA, JA / ET, ABA and MAPKs. Results suggest the possibility of a hijacking of the ABA signaling pathway by *A*. *lentis* which may lead to the suppression of the SA and JA / ET pathways; resulting in suppression of downstream defense responses. CDC Robin differed from the other two genotypes in its ability to negatively regulate ABA, possibly allowing it to significantly limit infection. This hypothesis needs to be confirmed in the future studies.

The identified candidate genes could be used for mapping and developing markers suitable for marker assisted selection. A recombinant inbred population derived from CDC Robin × 964a-46 has already been developed and was subjected to quantitative trait loci (QTL) mapping of ascochyta blight resistance [53,84]. Exploring the co-localization of candidate genes and the reported QTL using this population may reveal further evidence for the role of the candidate defense gene e.g. the NBS-LRR and LRK in ascochyta blight resistance. However, final confirmation for the role played by each of the candidate genes can only be provided by functional studies.

Results here suggest that 964a-46 and CDC Robin mediated ascochyta blight resistance through partially diverse mechanisms, making them ideal candidates for resistance gene pyramiding. Resistance mechanisms of CDC Robin also appear to differ from those of ILL 7537, hence ILL 7537 would represent another valuable resistance source for gene pyramiding. Moving forward, validating the role of candidate defense genes in resistance may help to design informative molecular markers suitable for marker-assisted selection to accelerate pyramiding the ascochyta blight resistance genes.

## Supporting information

S1 Table Number of plant-derived and pathogen-derived Illumina pair-ended reads. Reads were generated through RNA-sequencing of lentil genotypes Eston, CDC Robin and 964a-46 after *Ascochyta lentis* infection, and mapped to the *Lens culinaris* cv. CDC Redberry and *A*. *lentis* Al4 reference genomes.
(DOCX)

S2 Table Relative expression fold change of selected house-keeping genes. Fold changes were inferred from RNA-seq analysis in lentil genotypes Eston, CDC Robin and 964a-46 after inoculation with *Ascochyta lentis*. *GAPDH* = *Glyceraldehyde-3-Phosphate Dehydrogenase*, *TEF* = *Translation elongation factor*. Fold change in gene expression was calculated by Cuffdiff software by dividing fragments per kb of exon per million mapped reads (FPKM) value of infected samples by that of the non-infected sample (mock) sampled before inoculation. Relative expression fold change less than two was considered insignificant. Gene IDs were generated using Cufflink software and links data presented here to the transcript annotations in S1 File.
(DOCX)

S3 Table Number of differentially expressed genes in lentil genotypes. Lentil genotypes Eston, CDC Robin and 964a-46 were analyzed after *Ascochyta lentis* infection. Hpi, hours post inoculation. Relative expression fold change of two compared with the mock inoculated plants samples at time 0 was considered as a threshold for determining the differentially expressed genes.
(DOCX)

S4 Table Features of differentially expressed NBS-LRR genes. Sequence description, expression levels and genetic variants of nucleotide binding site-leucine rich repeat (NBS-LRR) genes in lentil genotypes Eston, CDC Robin and 964a-46 infected with *Ascochyta lentis*. Sequence descriptions are from the BLASTx against RefSeq release 60 hit with the highest percentage of sequence identity. Fold change in gene expression was calculated by Cuffdiff software by dividing fragments per kb of exon per million mapped reads (FPKM) value of infected samples to that of non-infected sample collected before inoculation (mock). Hpi = hours post inoculation with *Ascochyta lentis*. Gene IDs were generated using Cufflink software and links data presented here to the transcript annotations in S1 File.
(DOCX)

S5 Table Features of up-regulated candidate plant defense genes. Genes were up-regulated after *Ascochyta lentis* infection, but similarly expressed, in lentil genotypes Eston, CDC Robin and 964a-46. Sequence descriptions are from the BLASTx against RefSeq release 60 hit with the highest percentage of sequence identity. Gene symbols were extracted from the *Arabidopsis* information resource TAIR (http://www.arabidopsis.org). For genes with no gene symbol in TAIR, the abbreviation of sequence description was used. Fold change in gene expression was calculated by Cuffdiff software by dividing fragments per kb of exon per million mapped reads (FPKM) value of infected samples to that of non-infected sample collected before inoculation (mock). Hpi = hours post inoculation with *Ascochyta lentis*. Gene IDs were generated using Cufflink software and links data presented here to the transcript annotations in S1 File.
(DOCX)

S1 File Differentially expressed trasnscripts in lentil genotypes. The coordinates of transcripts differentially expressed in Eston, CDC Robin and 964a-46 upon *Ascochyta lentis* infection on the *Lens culinaris* CDC Redberry genome v. 0.6 (Lc v. 0.6) was reported for each transcript. The orthologues identified through BLASTing the CDC Redberry sequences against *Medicago trancatula* Mt 4.0 coding sequences (CDS), along with the percentage of sequence similarity and the E-value of the top BLAST hit are also reported for the transcripts.
(XLSX)

S2 File Genetic variants within differentially expressed NBS-LRR genes. Differentially expressed NBS-LRR genes of lentil genotypes Eston, CDC Robin and 964a-46 upon *Ascochyta lentis infection* with coordinates on the *Lens culinaris* CDC Redberry genome v. 0.6 (Lc v. 0.6).
(XLSX)
