# Supplementary material for: Defense responses of lentil (Lens culinaris) genotypes carrying non-allelic ascochyta blight resistance genes to Ascochyta lentis infection
Source: PLoS One. 2018 Sep 20;13(9):e0204124. doi: 10.1371/journal.pone.0204124 (PMC6147436; doi:10.1371/journal.pone.0204124)
Supplement: S1 Table — Reads were generated through RNA-sequencing of lentil genotypes Eston, CDC Robin and 964a-46 after Ascochyta lentis infection, and mapped to the Lens culinaris cv. CDC Redberry and A. lentis Al4 reference genomes. (DOCX) [file pone.0204124.s001.docx]

| Genotypes | Hpi | Total reads | Mapped to lentil genome | % | Uniquely mapped to lentil genome | % | Mapped to *A. lentis* genome | % |
| --- | --- | --- | --- | --- | --- | --- | --- | --- |
| Eston | 0 (mock) | 8,775,806 | 8,032,644 | 91.5 | 7,431,349 | 84.7 | 75 | - |
|  | 6 | 10,893,300 | 9,730,089 | 89.3 | 9,001,605 | 82.6 | 25,963 | 0.2 |
|  | 12 | 7,060,316 | 6,399,674 | 90.6 | 5,870,057 | 83.1 | 15,420 | 0.2 |
|  | 18 | 7,549,602 | 6,961,493 | 92.2 | 6,398,986 | 84.7 | 20,702 | 0.3 |
|  | 24 | 6,724,400 | 6,130,984 | 91.2 | 5,693,931 | 84.7 | 21,158 | 0.3 |
|  | 36 | 13,169,988 | 11,906,877 | 90.4 | 11,024,060 | 83.7 | 54,392 | 0.4 |
|  | 48 | 5,598,228 | 5,034,382 | 89.9 | 4,669,141 | 83.4 | 13,845 | 0.2 |
|  | 60 | 12,756,367 | 11,541,071 | 90.5 | 1,068,7367 | 83.8 | 26,039 | 0.2 |
|  | Average | 9,066,001 | 8,217,152 | 90.7 | 7,597,062 | 83.8 | 25,039 | 0.3 |
|  | Total | 72,528,007 | 65,737,214 | - | 6,077,6496 | - | 177,519 | - |
| CDC Robin | 0 (mock) | 7,525,507 | 6,836,741 | 90.8 | 6,347,415 | 84.3 | 31 | - |
|  | 6 | 15,013,507 | 13,685,136 | 91.2 | 12,570,779 | 83.7 | 24,057 | 0.2 |
|  | 12 | 6,286,050 | 5,702,624 | 90.7 | 5,274,034 | 83.9 | 8,498 | 0.1 |
|  | 18 | 6,307,549 | 5,688,398 | 90.2 | 5,188,774 | 82.3 | 14,957 | 0.2 |
|  | 24 | 7,948,399 | 7,505,332 | 94.4 | 6,686,352 | 84.1 | 24,995 | 0.3 |
|  | 36 | 6,722,494 | 6,084,783 | 90.5 | 5,594,456 | 83.2 | 24,456 | 0.4 |
|  | 48 | 7,340,280 | 6,615,538 | 90.1 | 6,115,844 | 83.3 | 24,953 | 0.3 |
|  | 60 | 5,887,879 | 5,293,015 | 89.9 | 4,839,965 | 82.2 | 11,133 | 0.2 |
|  | Average | 7,878,958 | 7,176,446 | 91.0 | 6,577,202 | 83.4 | 19,007 | 0.2 |
|  | Total | 63,031,665 | 57,411,567 | - | 52,617,619 | - | 133,049 | - |
| 964a-46 | 0 (mock) | 10,262,515 | 9,305,129 | 90.7 | 8,664,323 | 84.4 | 201 | - |
|  | 6 | 5,521,013 | 5,036,930 | 91.2 | 4,600,664 | 83.3 | 8,689 | 0.2 |
|  | 12 | 6,195,310 | 5,572,593 | 89.9 | 5,055,440 | 81.6 | 13,357 | 0.2 |
|  | 18 | 16,085,923 | 14,696,895 | 91.3 | 12,865,561 | 80.0 | 36,546 | 0.2 |
|  | 24 | 8,905,902 | 8,032,511 | 90.2 | 7,375,933 | 82.8 | 26,518 | 0.3 |
|  | 36 | 4,754,922 | 4,276,008 | 89.9 | 3,919,104 | 82.4 | 15,035 | 0.3 |
|  | 48 | 8,863,682 | 7,980,617 | 90.0 | 7,330,438 | 82.7 | 44,728 | 0.5 |
|  | 60 | 8,471,446 | 7,682,607 | 90.9 | 7,133,521 | 84.2 | 6,585 | 0.1 |
|  | Average | 8,632,589 | 7,822,911 | 90.5 | 7,118,123 | 82.7 | 21,637 | 0.3 |
|  | Total | 69,060,713 | 62,583,290 | - | 56,944,984 | - | 151,458 |  |
