# Supplementary material for: Defense responses of lentil (Lens culinaris) genotypes carrying non-allelic ascochyta blight resistance genes to Ascochyta lentis infection
Source: PLoS One. 2018 Sep 20;13(9):e0204124. doi: 10.1371/journal.pone.0204124 (PMC6147436; doi:10.1371/journal.pone.0204124)
Supplement: S2 Table — Fold changes were inferred from RNA-seq analysis in lentil genotypes Eston, CDC Robin and 964a-46 after inoculation with Ascochyta lentis. GAPDH = Glyceraldehyde-3-Phosphate Dehydrogenase, TEF = Translation elongation factor. Fold change in gene expression was calculated by Cuffdiff software by dividing fragments per kb of exon per million mapped reads (FPKM) value of infected samples by that of the non-infected sample (mock) sampled before inoculation. Relative expression fold change less than two was considered insignificant. Gene IDs were generated using Cufflink software and links data presented here to the transcript annotations in S1 File. (DOCX) [file pone.0204124.s002.docx]

| Genotype | Gene ID | Seq. description | Hours post inoculation | | | | | | |
| --- | --- | --- | --- | --- | --- | --- | --- | --- | --- |
|  |  |  | 6 | 12 | 18 | 24 | 36 | 48 | 60 |
| Eston | TSS10613 | *GAPDH* | -0.32 | 0.65 | 1.12 | 0.28 | 0.46 | 0.57 | 0.98 |
|  | TSS11673 | β*-tubulin* | -1.11 | -1.24 | 0.08 | -1.62 | -1.76 | -1.42 | -0.98 |
|  | TSS5364 | β*-tubulin* | 0.04 | -0.75 | -0.82 | -0.60 | -1.16 | -0.59 | -1.55 |
|  | TSS7557 | β*-tubulin* | -0.89 | -1.54 | -0.87 | -0.55 | -1.20 | -0.54 | -0.52 |
|  | TSS7835 | β*-tubulin* | -0.44 | -0.72 | -0.07 | -0.60 | -1.14 | -1.29 | -1.87 |
|  | TSS3436 | *DNAj Chaperon* | 0.08 | 0.13 | 0.16 | -0.11 | 0.28 | 0.10 | -0.11 |
|  | TSS468 | *DNAj Chaperon* | -0.26 | 0.05 | -0.78 | 0.51 | -0.49 | -0.48 | 0.65 |
|  | TSS9531 | *DNAj Chaperon* | 1.26 | 0.84 | 1.56 | 1.20 | 0.75 | 0.20 | -1.74 |
|  | TSS7880 | *TEF* | 1.17 | 1.39 | 1.27 | 0.78 | 1.03 | 1.41 | 0.86 |
|  | TSS8401 | *TEF* | 1.76 | 1.51 | 0.10 | 1.34 | 0.69 | 1.38 | 0.15 |
|  | TSS8566 | *TEF* | -0.59 | -0.42 | -1.26 | -1.67 | -0.34 | -0.20 | -0.14 |
| CDC Robin | TSS10613 | *GAPDH* | -0.58 | 0.01 | 0.27 | -0.17 | 0.56 | -0.07 | 0.01 |
|  | TSS11673 | β*-tubulin* | -0.90 | 0.89 | -1.50 | 0.05 | 0.85 | -0.58 | 0.63 |
|  | TSS5364 | β*-tubulin* | -0.20 | -0.69 | -1.70 | -1.74 | -1.08 | -1.26 | -1.5 |
|  | TSS7557 | β*-tubulin* | -1.68 | -1.14 | -1.10 | -1.24 | -0.86 | -1.04 | -0.33 |
|  | TSS7835 | β*-tubulin* | -0.79 | -0.88 | -0.18 | -1.44 | -0.83 | -0.93 | -2.74 |
|  | TSS3436 | *DNAj Chaperon* | 0.33 | 0.16 | 0.47 | 0.60 | 0.30 | 0.27 | -0.14 |
|  | TSS468 | *DNAj Chaperon* | 0.01 | -1.90 | 0.50 | 0.80 | -1.65 | -0.81 | 0.73 |
|  | TSS9531 | *DNAj Chaperon* | 1.01 | 1.08 | 0.45 | 0.20 | 0.35 | 1.40 | -1.02 |
|  | TSS7880 | *TEF* | -0.29 | 0.91 | 0.77 | 1.30 | 1.08 | 0.61 | 0.11 |
|  | TSS8401 | *TEF* | -0.51 | -0.67 | -1.06 | -0.13 | -0.51 | -0.21 | -2.14 |
|  | TSS8566 | *TEF* | -1.47 | -0.62 | -0.96 | -0.03 | -0.56 | -0.50 | -0.22 |
| 964a-46 | TSS10613 | *GAPDH* | -0.39 | 0.26 | 0.14 | 0.13 | -0.46 | -0.39 | 1.22 |
|  | TSS11673 | β*-tubulin* | 0.41 | 0.42 | 0.35 | 1.89 | 0.97 | 0.02 | 0.07 |
|  | TSS5364 | β*-tubulin* | 0.67 | -0.19 | -0.65 | -0.69 | -1.34 | -1.50 | 0.67 |
|  | TSS7557 | β*-tubulin* | -0.78 | -0.86 | -1.17 | -1.67 | -1.01 | -1.24 | 0.33 |
|  | TSS7835 | β*-tubulin* | -0.63 | -0.43 | -1.02 | -1.14 | -0.96 | -1.02 | -1.56 |
|  | TSS3436 | *DNAj Chaperon* | -0.02 | -0.09 | 0.08 | 0.25 | 0.36 | 0.04 | 2.23 |
|  | TSS468 | *DNAj Chaperon* | 0.20 | 0.17 | 0.38 | 0.80 | 0.40 | -0.55 | 0.02 |
|  | TSS9531 | *DNAj Chaperon* | 0.28 | 0.24 | 0.50 | 0.71 | 0.77 | 0.43 | 0.12 |
|  | TSS7880 | *TEF* | 0.13 | 0.24 | 0.14 | 1.19 | 1.17 | 1.01 | -0.08 |
|  | TSS8401 | *TEF* | 0.23 | -0.18 | -0.55 | 0.98 | -0.94 | -0.32 | 0.37 |
|  | TSS8566 | *TEF* | -1.09 | -0.95 | -0.74 | -0.30 | -0.94 | -0.48 | -0.36 |
