# Supplementary material for: Defense responses of lentil (Lens culinaris) genotypes carrying non-allelic ascochyta blight resistance genes to Ascochyta lentis infection
Source: PLoS One. 2018 Sep 20;13(9):e0204124. doi: 10.1371/journal.pone.0204124 (PMC6147436; doi:10.1371/journal.pone.0204124)
Supplement: S3 Table — Lentil genotypes Eston, CDC Robin and 964a-46 were analyzed after Ascochyta lentis infection. Hpi, hours post inoculation. Relative expression fold change of two compared with the mock inoculated plants samples at time 0 was considered as a threshold for determining the differentially expressed genes. (DOCX) [file pone.0204124.s003.docx]

| Genotype | hpi | UP-regulated | Down-regulated |
| --- | --- | --- | --- |
| Eston | 6 | 1018 | 1116 |
|  | 12 | 2439 | 1651 |
|  | 18 | 1080 | 1321 |
|  | 24 | 1288 | 1362 |
|  | 36 | 1633 | 2744 |
|  | 48 | 1338 | 2209 |
|  | 60 | 1104 | 2034 |
| CDC Robin | 6 | 1530 | 1564 |
|  | 12 | 1669 | 2245 |
|  | 18 | 1716 | 1997 |
|  | 24 | 1861 | 3022 |
|  | 36 | 1520 | 2781 |
|  | 48 | 1486 | 2665 |
|  | 60 | 1035 | 1928 |
| 964a-46 | 6 | 1291 | 1315 |
|  | 12 | 1113 | 1840 |
|  | 18 | 978 | 1937 |
|  | 24 | 1870 | 3130 |
|  | 36 | 1567 | 2475 |
|  | 48 | 1665 | 3118 |
|  | 60 | 1177 | 2029 |
