# Supplementary material for: Defense responses of lentil (Lens culinaris) genotypes carrying non-allelic ascochyta blight resistance genes to Ascochyta lentis infection
Source: PLoS One. 2018 Sep 20;13(9):e0204124. doi: 10.1371/journal.pone.0204124 (PMC6147436; doi:10.1371/journal.pone.0204124)
Supplement: S4 Table — Sequence description, expression levels and genetic variants of nucleotide binding site-leucine rich repeat (NBS-LRR) genes in lentil genotypes Eston, CDC Robin and 964a-46 infected with Ascochyta lentis. Sequence descriptions are from the BLASTx against RefSeq release 60 hit with the highest percentage of sequence identity. Fold change in gene expression was calculated by Cuffdiff software by dividing fragments per kb of exon per million mapped reads (FPKM) value of infected samples to that of non-infected sample collected before inoculation (mock). Hpi = hours post inoculation with Ascochyta lentis. Gene IDs were generated using Cufflink software and links data presented here to the transcript annotations in S1 File. (DOCX) [file pone.0204124.s004.docx]

| Gene ID | Gene name | Sequence description | Peak time (hpi) | Log_2_ fold change | Domains (number of SNPs) |  |
| --- | --- | --- | --- | --- | --- | --- |
|  | **Eston** | | | |  |  |
| TSS27158 | *RGA12* | nbs resistance protein | 12 | 14.5 | NBS (2), LRR (1), other (8) |  |
| TSS2124 | *RGA14* | tmv resistance protein n-like | 12 | 13.0 | TIR (0), LRR (0), other (3) |  |
| TSS27159 | *RGA17* | disease resistance rpp13-like protein | 12 | 4.8 | NBS (6), other (23) |  |
| TSS3519 | *RGA5* | tmv resistance protein n-like | 18 | 10.5 | TIR(0), LRR (0), other (3) |  |
| TSS19614 | *RGA21* | cc-nbs-lrr resistance protein | 18 | 13.0 | CC (0), NBS(4), other (0) |  |
| TSS10683 | *RGA4* | cc-nbs-lrr resistance protein | 24 | 11.0 | NBS (2), other (6) |  |
| TSS23730 | *RGA24* | nbs resistance protein | 24 | 12.6 | TIR (1), NBS (0), other (6) |  |
| TSS6388 | *RGA25* | cc-nbs-lrr resistance protein | 24 | 10.3 | NBS (1), LRR (0), other (3) |  |
| TSS17198 | *RGA27* | nbs-lrr resistance protein | 24 | 10.1 | NBS (3), LRR (1), other (8) |  |
| TSS25883 | *RGA1* | nbs-containing resistance-like protein | 36 | 13.9 | NBS (1), LRR (2), other (14) |  |
| TSS7773 | *RGA26* | resistance protein | 36 | 10.8 | TIR (0), NBS (0), other (5) |  |
| TSS1506 | *RGA30* | nbs resistance protein | 36 | 11.0 | TIR (0), NBS (0), other (1) |  |
| TSS19622 | *RGA35* | tmv resistance protein n-like | 48 | 12.2 | TIR (0), NBS (0), other (0) |  |
|  | **CDC Robin** | | | |  |  |
| TSS20657 | *RGA43* | nbs-containing resistance-like protein | 18 | 11.7 | CC (0), NBS (1), LRR (0), other (0) |  |
| TSS8718 | *RGA49* | tir-nbs-lrr disease resistance protein | 24 | 10.9 | TIR (0), LRR (0), other (23) |  |
| TSS17017 | *RGA41* | disease resistance rpp13-like protein | 48 | 15.3 | NBS (3), LRR (2), Other (8) |  |
| TSS17687 | *RGA48* | tir-nbs-lrr disease resistance protein | 48 | 15.3 | ZF (1), TIR (0), NBS (0), other (1) |  |
|  | **964a-46** | | | |  |  |
| TSS6621 | *RGA56* | tir-nbs-lrr resistance protein | 12 | 14.8 | NA |  |
| TSS12666 | *RGA68* | resistance protein | 12 | 15.6 | TIR (0), LRR (0), other (1) |  |
| TSS26294 | *RGA69* | nbs-lrr resistance protein | 18 | 12.4 | NA |  |
| TSS23628 | *RGA60* | resistance protein | 36 | 13.0 | CC (1), NBS (0), other (1) |  |
| TSS23225 | *RGA63* | cc-nbs-lrr resistance protein | 48 | 4.6 | NA |  |
| TSS23279 | *RGA58* | disease resistance protein rpm1-like | 60 | 11.5 | NA |  |
| TSS13487 | *RGA64* | disease resistance-like protein | 60 | 13.9 | NA |  |
| TSS15293 | *RGA71* | tmv resistance protein n-like | 48 | 13.7 | NA |  |
|  | **Eston & CDC Robin** | | | |  |  |
| TSS7950 | *RGA3*  Eston | tir-nbs-lrr resistance protein | 24 | 13.0 | NA |  |
|  | CDC Robin |  | 60 | -7.8 |  |  |
| TSS25528 | *RGA8*  Eston | nbs-lrr type disease resistance protein | 48 | 7.5 | CC (1), NBS (1), other (9) |  |
|  | CDC Robin |  | 36 | -14.1 |  |  |
| TSS23940 | *RGA28*  Eston | tmv resistance protein n-like | 24 | 2.8 | TIR (1), LRR (0), other (5) |  |
|  | CDC Robin |  | 48 | 9.6 |  |  |
| TSS7615 | *RGA32*  Eston | tmv resistance protein n | 36 | 2.5 | NA |  |
|  | CDC Robin |  | 12 | 11.8 |  |  |
| TSS23593 | *RGA34*  Eston | disease resistance protein | 48 | 14.5 | NA |  |
|  | CDC Robin |  | 24 | 14.5 |  |  |
|  | **Eston & 964a-46** | | | |  |  |
| TSS16763 | *RGA6*  Eston | tmv resistance protein n-like | 6 | 9.1 | TIR (0). LRR (0), other (1) |  |
|  | 964a-46 |  | 60 | -11.5 |  |  |
| TSS5710 | *RGA19*  Eston | tir-nbs-lrr rct1-like resistance protein | 48 | -13.5 | TIR(0), NBS (0), TIR (0), other (6) |  |
|  | 964a-46 |  | 12 | 11.2 |  |  |
| TSS4622 | *RGA22*  Eston | resistance protein | 18 | 12.4 | TIR (0), Other (6) |  |
|  | 964a-46 |  | 18 | 14.4 |  |  |
| TSS15293 | *RGA23*  Eston | tmv resistance protein n-like isoform | 24 | 13.8 | NA |  |
|  | 964a-46 |  | 24 | 2.8 |  |  |
| TSS14629 | *RGA37*  Eston | cc-nbs resistance protein | 48 | -16.0 | NBS(0), LRR (1), other (5) |  |
|  | 964a-46 |  | 18 | 8.2 |  |  |
|  | **CDC Robin & 964a-46** | | | |  |  |
| TSS26091 | *RGA45*  CDC Robin | cc-nbs-lrr resistance protein | 24 | 11.1 | NBS(0), LRR (3), other (4) |  |
|  | 964a-46 |  | 12 | 12.1 |  |  |
| TSS14643 | *RGA50*  CDC Robin | nbs-lrr resistance protein | 36 | 11.2 | TIR(0), NBS (0), other (1) |  |
|  | 964a-46 |  | 48 | 8.0 |  |  |
|  | **Eston &CDC Robin & 964a-46** | | | |  |  |
| TSS26091 | *RGA11*  Eston | cc-nbs-lrr resistance protein | 6 | -12.0 | NBS(0), LRR (1), other (7) |  |
|  | CDC Robin |  | 24 | 11.1 |  |  |
|  | 964a-46 |  | 12 | 12.1 |  |  |
| TSS1744 | *RGA13*  Eston | nbs-lrr type disease resistance protein | 12 | 13.5 | CC(2), NBS(0), LRR (1), other (3) |  |
|  | CDC Robin |  | 48 | 10.4 |  |  |
|  | 964a-46 |  | 12 | 9.3 |  |  |
