# Supplementary material for: Defense responses of lentil (Lens culinaris) genotypes carrying non-allelic ascochyta blight resistance genes to Ascochyta lentis infection
Source: PLoS One. 2018 Sep 20;13(9):e0204124. doi: 10.1371/journal.pone.0204124 (PMC6147436; doi:10.1371/journal.pone.0204124)
Supplement: S5 Table — Genes were up-regulated after Ascochyta lentis infection, but similarly expressed, in lentil genotypes Eston, CDC Robin and 964a-46. Sequence descriptions are from the BLASTx against RefSeq release 60 hit with the highest percentage of sequence identity. Gene symbols were extracted from the Arabidopsis information resource TAIR (http://www.arabidopsis.org). For genes with no gene symbol in TAIR, the abbreviation of sequence description was used. Fold change in gene expression was calculated by Cuffdiff software by dividing fragments per kb of exon per million mapped reads (FPKM) value of infected samples to that of non-infected sample collected before inoculation (mock). Hpi = hours post inoculation with Ascochyta lentis. Gene IDs were generated using Cufflink software and links data presented here to the transcript annotations in S1 File. (DOCX) [file pone.0204124.s005.docx]

|  | Sequence description | Gene symbol | Genotypes | | | | | |
| --- | --- | --- | --- | --- | --- | --- | --- | --- |
| Gene ID |  |  | Eston | | CDC Robin | | 964a-46 | |
|  |  |  | Peak time (hpi) | Log_2_ fold change | Peak time (hpi) | Log_2_ fold change | Peak time (hpi) | Log_2_ fold change |
| TSS5598 | abscisic acid receptor pyl6 | *Pyl6* | 6 | 5.7 | 12 | 6.3 | 24 | 7.7 |
| TSS22528 | peroxidase | *POX* | 6 | 3.4 | 6 | 3.6 | 60 | 3.1 |
| TSS7712 | cysteine-rich receptor like kinase | *CRRLK* | 6 | 4.2 | 6 | 5.6 | 6 | 4.0 |
| TSS6447 | chalcone synthase | *CHS* | 12 | 5.2 | 6 | 5.7 | 6 | 3.8 |
| TSS8207 | peroxidase 12-like | *POX12* | 12 | 7.6 | 18 | 6.8 | 24 | 7.4 |
| TSS9306 | glutathione s-transferase gstu6-like | *GSTU6* | 12 | 6.1 | 6 | 5.3 | 12 | 5.4 |
| TSS7567 | pathogenesis-related protein STH2 | *PR-10* | 12 | 4.7 | 6 | 4.2 | 6 | 3.3 |
| TSS4884 | aba-responsive protein abr17 | *PR-10* | 12 | 6.9 | 18 | 7.6 | 12 | 5.5 |
| TSS14900 | pathogenesis-related protein pr10 | *PR-10* | 12 | 9.0 | 18 | 9.1 | 24 | 9.9 |
| TSS25036 | linoleate 13s-lipoxygenase | *LOX* | 12 | 7.1 | 6 | 5.9 | 12 | 5.5 |
| TSS10659 | acidic chitinase | *CHI* | 12 | 6.7 | 12 | 5.9 | 6 | 7.5 |
| TSS6091 | endo-beta-1 3-glucanase | *BG* | 12 | 5.7 | 18 | 6.7 | 18 | 5.8 |
| TSS12817 | isoflavone reductase | *IR* | 12 | 5.6 | 6 | 5.1 | 12 | 3.7 |
| TSS27064 | 2-succinylbenzoate-CoA ligase | *SBCL* | 12 | 4.2 | 12 | 3.3 | 24 | 4.3 |
| TSS27086 | guanine nucleotide-binding protein | *GP* | 12 | 3.6 | 18 | 4.7 | 12 | 3.9 |
| TSS17013 | cellulose synthase-like protein e1 | *CSL* | 12 | 3.7 | 12 | 4.0 | 24 | 2.8 |
| TSS893 | pleiotropic drug resistance protein 3-like | *PDR* | 12 | 3.7 | 6 | 4.0 | 6 | 2.1 |
| TSS7765 | disease resistance response protein pi49 | *PR-10* | 12 | 7.2 | 6 | 6.9 | 6 | 5.5 |
| TSS4253 | flavonoid 3 -monooxygenase-like | *FOX* | 18 | 3.0 | 48 | 3.4 | 36 | 2.1 |
| TSS21476 | peroxidase 53-like | *POX-53* | 24 | 6.1 | 48 | 4.5 | 18 | 5.0 |
| TSS9305 | flavonoid 3 -hydroxylase | *FH* | 24 | 3.4 | 24 | 2.1 | 48 | 1.4 |
| TSS26152 | snf1-related protein kinase | *SNRK* | 48 | 5.6 | 24 | 5.5 | 24 | 6.3 |
| TSS18562 | peroxidase 4-like | *POX4* | 12 | 4.4 | 6 | 5.3 | 6 | 4.3 |
| TSS6275 | somatic embryogenesis receptor kinase | *SERK* | 6 | 3.4 | 6 | 4.0 | 6 | 2.9 |
| TSS24222 | receptor-like serine threonine kinase fls2 | *FLS2* | 6 | 3.7 | 6 | 3.4 | 6 | 3.0 |
| TSS17818 | phenylalanine ammonia-lyase class 3- like | *PAL* | 6 | 3.7 | 6 | 3.2 | 6 | 1.9 |
| TSS12956 | alcohol dehydrogenase-like 5-like | *ADH* | 12 | 4.3 | 6 | 4.5 | 6 | 2.9 |
| TSS21085 | f-box protein pp2-a13-like | *PP2-A13* | 36 | 3.7 | 6 | 3.5 | 24 | 5.6 |
| TSS2005 | cytosolic fructose-1 6-bisphosphatase | *FBP* | 12 | 4.6 | 24 | 4.9 | 24 | 7.1 |
| TSS13544 | ethylene receptor 2-like | *ERL* | 36 | 3.8 | 48 | 4.3 | 6 | 2.7 |
| TSS23249 | auxin response factor | *ARF* | 60 | 13.9 | 18 | 13.5 | 12 | 13.8 |
| TSS5684 | potassium transporter 5-like | *KUP* | 12 | 5.8 | 18 | 6.4 | 24 | 5.6 |
| TSS16156 | wrky transcription factor 51-like | *WRKY-51* | 36 | 2.9 | 36 | 3.2 | 36 | 3.1 |
| TSS11145 | ethylene-overproduction protein 1-like | *ETO1* | 36 | 3.3 | 24 | 4.0 | 24 | 4.4 |
